# Persistent Mayer Dirac

**DOI:** 10.1088/2632-072X/ad83a5

**Published:** 2024-10-17

**Authors:** Faisal Suwayyid, Guo-Wei Wei

**Affiliations:** 1 Department of Mathematics, King Fahd University of Petroleum and Minerals, Dhahran 31261, Saudi Arabia; 2 Department of Mathematics, Michigan State University, East Lansing, MI 48824, United States of America; 3 Department of Electrical and Computer Engineering, Michigan State University, East Lansing, MI 48824, United States of America; 4 Department of Biochemistry and Molecular Biology, Michigan State University, East Lansing, MI 48824, United States of America

**Keywords:** N-chain complex, Mayer homology, Mayer Laplacian, Mayer Dirac, persistent homology, topological signals, biology modeling

## Abstract

Topological data analysis (TDA) has made significant progress in developing a new class of fundamental operators known as the Dirac operator, particularly in topological signals and molecular representations. However, the current approaches being used are based on the classical case of chain complexes. The present study establishes Mayer Dirac operators based on *N*-chain complexes. These operators interconnect an alternating sequence of Mayer Laplacian operators, providing a generalization of the classical result $D^2 = L$. Furthermore, the research presents an explicit formulation of the Laplacian for *N*-chain complexes induced by vertex sequences on a finite set. Weighted versions of Mayer Laplacian and Dirac operators are introduced to expand the scope and improve applicability, showcasing their effectiveness in capturing physical attributes in various practical scenarios. The study presents a generalized version for factorizing Laplacian operators as an operator’s product and its ‘adjoint’. Additionally, the proposed persistent Mayer Dirac operators and extensions are applied to biological and chemical domains, particularly in the analysis of molecular structures. The study also highlights the potential applications of persistent Mayer Dirac operators in data science.

## 1. Introduction

Topological Data Analysis (TDA) has emerged as a rapidly growing field, owing to the advances in machine learning techniques, improved computational power, and the abundance of data with varying dimensions and complexities [4, 8, 30, 34, 35]. The discipline has found applications in multiple domains such as physics, biology, chemistry, engineering, and computer science [19, 45, 49, 60]. The initial success of TDA is mainly due to persistent homology [16, 28, 29, 67], a technique integrating topology and multiscale analysis. In the beginning, persistent homology was used to give qualitative insights about the structure and shape of data. Subsequently, persistent homology was utilized in quantitative prediction [1, 65, 66]. Sophisticated machine learning algorithms enable persistent homology to be applied to large-scale data [13]. Topological deep learning (TDL) was coined in 2017 [15], which has emerged as a new paradigm in data science [50]. Some of the most impressive achievements of TDL include its dominant victories in D3R Grand Challenges, a worldwide competition series in computer-aided drug design [47, 48], the discovery of two SARS-CoV-2 evolutionary mechanisms [22, 61], and the successful forecasting of SARS-CoV-2 new variants BA.2 [23] and BA.4/BA.5 [21] about two months in advance.

Technically, persistent homology helps track and capture topological properties across different scales in a dataset. This approach creates a sequence of topological spaces through filtration, capturing the topological characteristics and evolution of features across scales in contrast to traditional static data analysis methods. By using data filtration, persistent homology recognizes enduring patterns, uncovering persistent topological signatures that conventional approaches may overlook. This innovative approach enhances the strength and depth of insights gained from TDA, making it a valuable methodology for unraveling complex relationships within diverse datasets. Researchers have expanded their exploration beyond single persistent homology, venturing into multi-persistent homology [18], Zig-zag persistent homology [17], Cayley persistent homology [6], and numerous other variants. However, persistent homology has many limitations, particularly its inability to portray features beyond topological invariants and to embed quantitative and localized information. Wei and his colleagues introduced persistent Laplacians on the point cloud, known as Persistent Spectral Graph (PSG) or persistent combinatorial Laplacian [62], and on the differentiable manifold, known as evolutionary de Rham-Hodge method [24], as a remedy for the limitations associated with persistent homology. These operators encapsulate both the harmonic portion of the spectrum of the persistent Laplacian operator, which is the same as that derived from persistent homology, and the non-harmonic part, which imparts geometric insights into the simplicial complex. The advantages of persistent Laplacians over persistent homology were examined by using 34 datasets in protein engineering [53]. Their outstanding performance is evident in their ability to predict the emergence of dominant viral variants effectively [21].

Notably, the evolutionary de Rham-Hodge theory was formulated to integrate multiscale analysis into the traditional de Rham-Hodge theory on manifolds, creating persistent Hodge Laplacians [24]. However, the application of this advanced theory to Riemannian manifolds presents a significant computational challenge. The PSG approach involves transforming a point cloud into a sequence of simplicial complexes generated through filtration [62]. Its harmonic spectra align with the topological persistence observed in persistent homology. In contrast, the non-harmonic spectra play a crucial role in capturing the homotopic shape evolution of the data during the filtration process. Mémoli *et al* provided an effective computational algorithm for persistent Laplacians [44]. The stability of persistent Laplacians was analyzed by Liu *et al* [39]. Various persistent Laplacian approaches have been devised, each excelling in specific aspects [33, 39, 56, 63, 64]. For example, the Persistent Sheaf Laplacian (PSL) [64] aims to enhance the handling of point cloud data with labeled information and facilitate multiscale analysis. This method extends the framework of cellular sheaves, enabling the incorporation of non-geometric information into the topological invariants and spectral representations [31, 57]. A novel approach was developed that considers directional information, applying filtration to path complexes derived from directed graphs [63]. Addressing a similar limitation, the persistent hyperdigraph Laplacian was introduced [20]. Both approaches account for directional relations, with hyperdigraph homology and persistent hyperdigraph homology focusing on capturing more intricate directional information in the data.

Recently, the field of TDA has witnessed significant strides in developing a new class of fundamental operators known as Dirac [2]. These operators serve as formal square roots of Laplacian operators, and they encapsulate information similar to Laplacian operators while providing additional insights into subchain complexes. One of their key attributes is their ability to represent the homologies of their complexes as distinct subspaces within their kernels, which enhances their ability to detect subtle topological changes during different phases of data filtration [2, 5, 35]. Dirac operators are instrumental in analyzing topological signals. They are particularly useful in revealing unique properties such as explosive behavior and hysteresis loops in Dirac synchronization on fully connected networks [10, 12]. Moreover, Dirac operators find applications in developing Dirac signal processing techniques that filter noisy topological signals defined on various elements of simplicial complexes, such as nodes, links, and triangles [9, 11, 12]. A computational framework has been established for molecular representation, utilizing the concept of the persistent Dirac operator [4, 32, 35]. This framework systematically explores the properties of the spectrum of discrete Dirac matrices, examining how different weighting schemes impact the information encoded in Dirac eigenspectra, thereby providing deeper insights into molecular structures. Persistent Path Dirac operators have been introduced to account for directional information and higher relations, utilizing path complexes of digraphs and hypergraphs [59]. This proves valuable in representing molecular interactions between atoms and systematically capturing insights into molecular structures. However, it is essential to highlight that all the formulations mentioned above are constructed based on the development of chain complexes. The *N*-chain complex exhibits characteristics that differ from those of typical chain complexes [26, 27, 36, 40, 41, 43, 58]. Shen *et al* introduced persistent Mayer homology and persistent Mayer Laplacians specifically tailored for *N*-chain complexes to address this distinction [56]. Mayer’s homology may not necessarily yield a homotopy invariant, but persistent Mayer homology and persistent Mayer Laplacians can reflect specific geometric structures and additional topological features of simplicial complexes. This emphasizes persistent Mayer homology’s robust capability in characterizing geometric and topological aspects. Furthermore, the computation of persistent Mayer homology proves to be significantly faster than computing the usual Laplacian, highlighting a distinct advantage of persistent Mayer homology [56].

To explore the Mayer *N*-chain complexes and Mayer homologies in greater detail, this study revisits the definitions of Mayer *q*th *n*-cycles, *q*th *n*-boundaries, and Mayer homology groups. Classical examples are revisited, and a table detailing non-zero Mayer homological groups linked to initial simplicies is provided. Subsequently, the study revisits the fundamental outcomes of Laplacians and Dirac operators within classical chain complexes, exemplifying their algebraic relations and properties, particularly in the context of 2-chain complexes. Having established this groundwork, the definitions of *q*th Mayer Laplacians are recalled, Mayer Dirac operators are established, and their significance is demonstrated. These operators interconnect an alternating sequence of *q* and *N* − *q* Mayer Laplacian operators and offer a generalization of the classical result $D^2 = L$. Specifically, the equivalence between the square of the product of Mayer Dirac operators and the Mayer Laplacians is established. Moreover, the study presents an explicit formulation of the Laplacian for *N*-chain complexes induced by vertex sequences on a finite set *V*. Notably, these formulas simplify when applied to simplicial complexes. Weighted versions of Laplacian and Dirac operators are introduced to broaden the scope and enhance applicability, showcasing their efficacy in capturing physical attributes in various practical scenarios. Inspired by classical Dirac operators, a generalized version for factorizing Laplacian operators as an operator’s product and its ‘adjoint’ is presented. Additionally, the study delves into the realm of persistent Mayer Laplacian operators, defining persistent Mayer Dirac operators. Extending the research to biological and chemical domains, particularly in the study of molecular structures, the study highlights the application potential of persistent Mayer Dirac operators. Several potential applications of Mayer Dirac operators are proposed, demonstrating their superior insights compared to classical counterparts. Moreover, the study suggests utilizing Mayer Dirac operators as fingerprints, emphasizing their ability to capture broader distinctions between two molecules. Finally, the study proposes further research on Mayer Dirac operators to highlight their untapped potential in various domains.

## 2. Preliminaries

Mayer homology is an extension of homologies that use boundary operators *d* with the property of *d*^2^ = 0. It is constructed using a sequence of abelian groups $C_* = (C_n)_{n\unicode{x2A7E} 0}$ that are connected by boundary operators $(d_n: C_n\to C_{n-1})_{n\unicode{x2A7E} 1}$, where *d*_0_ = 0, such that by abuse of notation, $d^N = 0$ for some $N\unicode{x2A7E} 2$, while *d*^2^ may not be equal to zero. This allows for comparing higher-degree components in the sequence with those of lower degrees. In general, an arbitrary field is denoted by $\mathbb{K}$; however, this article focuses on the scenario where $C_*$ is a sequence over the complex field $\mathbb{K} = \mathbb{C}$, and it is assumed that $N\unicode{x2A7E} 2$. Therefore, if not stated otherwise, it is assumed that $\mathbb{K} = \mathbb{C}$ throughout the article.

### 2.1. Mayer homology

Definition 2.1. An *N-chain complex* is defined as a sequence of $\mathbb{K}$-linear spaces $C_{\ast} = (C_{n})_{n\unicode{x2A7E} 0}$, accompanied by a sequence of linear maps $d = (d_n: C_n \to C_{n-1})$. These maps, referred to as the *N-differentials (N-boundary operators)*, satisfy the condition $d_nd_{n+1}\cdots d_{n+N-2}d_{n+N-1} = 0$ for every integer $n\unicode{x2A7E} 1$.

When *N* = 2, the structure reduces to that of a normal chain complex. Let $(C_*,d)$ denote an *N*-chain complex. To simplify notation, we introduce the shorthand

\begin{align*}d_{n}^t: = d_{n-\left(t-1\right)}\cdots d_{n-1}d_n,\end{align*}

where *t* is a positive integer and *n* is a non-negative integer. Consequently, $d_n^t :C_n \rightarrow C_{n-t}$, and if $t\unicode{x2A7E} N$, then $d_n^t = 0$. For each $1\unicode{x2A7D} q\unicode{x2A7D} N-1$, the *qth n-cycles* are defined as

\begin{align*}Z_{n,q} = \ker\left(d_n^q\right) = \left\{x\in C_{n}|d_n^{q}x = 0\right\},\end{align*}

and the *qth n-boundaries* are given by

\begin{align*}B_{n,q} = \textrm{im}\left(d_{n+N-q}^{N-q}\right) = \left\{d_{n+N-q}^{N-q}x|x\in C_{n+N-q}\right\}.\end{align*}

Notably, $B_{n,q}\subseteq Z_{n,q}$ due to the identity $0 = d_{n+N-q}^N = d_n^qd_{n+N-q}^{N-q}$. The *Mayer homology groups* of the *N*-chain complex $(C_{\ast},d)$ are defined as

\begin{align*} H_{n,q}\left(C_{\ast},d\right): = Z_{n,q}/B_{n,q},\quad n\unicode{x2A7E} 0, 1\unicode{x2A7D} q\unicode{x2A7D} N-1.\end{align*}

Simplified notations like $H_{n,q}$ or $H_{n,q}(C)$ are employed when no ambiguity arises. The *Mayer Betti number* of the *N*-chain complex $(C_{\ast},d)$, denoted by $\beta_{n,q}$, is defined as the dimension of $H_{n,q}(C_{\ast},d)$. It is important to note that $Z_{n,q} = \ker(d_n^q)$ and $B_{n-q, N-q} = \textrm{Im}(d_n^q)$. Utilizing the exactness of the sequences

\begin{align*} 0 \xrightarrow[]{~~~~~~} Z_{n,q} \xrightarrow[]{~~~~~~} C_{n} \xrightarrow[~~~~~~]{{{d_{n}^q}}} B_{n-q,N-q} \xrightarrow[]{~~~~~~} 0,\end{align*}

and

\begin{align*} 0 \xrightarrow[]{~~~~~~} B_{n,q} \xrightarrow[]{~~~~~~} Z_{n,q} \xrightarrow[]{~~~~~~} H_{n,q} \xrightarrow[]{~~~~~~} 0,\end{align*}

it follows that

\begin{align*} \dim H_{n,q}\left(C\right)&amp; = \dim Z_{n,q} - \dim B_{n,q}\nonumber\\ &amp;= \dim C_n - \dim B_{n-q,N-q}-\dim B_{n,q}\nonumber\\ &amp;= \dim C_n - {\mathrm{rank}\hspace{0.1em}}\left(d_n^q\right)-{\mathrm{rank}\hspace{0.1em}} \left(d_{n+N-q}^{N-q}\right).\end{align*}

Considering $(C_*, d)$ as a differential graded $\mathbb{K}$-linear space of degree −1, e.g. setting $\widehat{C}_* = \bigoplus_{n\unicode{x2A7E} 0}C_n$, it is noteworthy that $(C_*, d)$ is an *N*-chain complex if and only if the map induced by *d* on $\widehat{C}_*$, denoted as $\widehat{d}: \widehat{C}_* \to \widehat{C}_*$, satisfies $\widehat{d}^{\: N} = 0$. This formulation is frequently employed in constructing Laplacian and Dirac operators.

Example 1. Let *p* be a prime integer, and $\mathbb{K} = \mathbb{Z}_p$. Consider the polynomial ring over $\mathbb{Z}_p$, $\mathbb{Z}_{p}[x]$, and set $C_n = \mathbb{Z}_{p}[x]$ for $n\unicode{x2A7E} 0$. Consider the linear map $d:\mathbb{Z}_{p}[x]\to \mathbb{Z}_{p}[x]$ defined by $dx^{n} = nx^{n-1}$ for $n\unicode{x2A7E} 1$, and $d(1) = 0$. Let $n\unicode{x2A7E} 1$, and $m\unicode{x2A7E} 0$. If *m* < *n*, then an inductive argument shows that $d^nx^m = m!d^{n-m}(1) = 0$. If, on the other hand, $m\unicode{x2A7E} n$, then another inductive argument shows that

\begin{align*} d^{n}x^m = m\left(m-1\right)\left(m-2\right)\cdots \left(m-\left(n-1\right)\right)x^{m-n}.\end{align*}

Consequently, $d^nx^m = 0$ if and only if *p* divides *m* − *r* for some $0\unicode{x2A7D} r\unicode{x2A7D} n-1$. This condition holds if and only if $m = kp+r$ for some $k\unicode{x2A7E} 0$ and $0\unicode{x2A7D} r < n$. Therefore, $d^n = 0$ if $n\unicode{x2A7E} p$, specifically $d^p = 0$. If *n* < *p*, then

\begin{align*}\ker d^n = \textrm{span}\left(\left\{x^m| m = kp+r, 0\unicode{x2A7D} r < n, k\unicode{x2A7E} 0\right\}\right).\end{align*}

By following analogous steps, it is derived that

\begin{align*}\textrm{Im }\ d^n = \textrm{span}\left(\left\{x^m| m = kp+r, 0\unicode{x2A7D} r < p-n, k\unicode{x2A7E} 0\right\}\right)\end{align*}

for $p > n\unicode{x2A7E} 1$. Let $d_n :C_n \to C_{n-1}$ be equal to *d* for all $n\unicode{x2A7E} 1$. Thus, $(C_*, d)$ forms a *p*-chain, and for $1\unicode{x2A7D} q \unicode{x2A7D} p-1$, we have

\begin{align*}Z_{n,q} = \ker d^q = \textrm{span}\left(\left\{x^m| m = kp+r,0\unicode{x2A7D} r\unicode{x2A7D} q-1, k\unicode{x2A7E} 0\right\}\right),\end{align*}

\begin{align*}B_{n,q} = \textrm{Im }\ d^{p-q} = \textrm{span}\left(\left\{x^m| m = kp+r,0 \unicode{x2A7D} r\unicode{x2A7D} q-1, k\unicode{x2A7E} 0\right\}\right).\end{align*}

According to the definition, the Mayer homology is given by

\begin{align*} H_{n,q}\left(C_{\ast},d\right) = 0, \quad n\unicode{x2A7E} 0.\end{align*}

Example 2. In this example, we will assume that $\mathbb{K} = \mathbb{C}$. We will use a construction that involves the primitive *N*th root of unity, denoted by $\xi = e^{2\pi \sqrt{-1}/N}$. This construction will be used frequently, so it is essential to understand it well. For $k\unicode{x2A7E} 1$, we have the identity:

\begin{align*}\sum\limits_{i = 0}^{k-1}\xi^{i} = \frac{\xi^k-1}{\xi -1}.\end{align*}

It is then apparent that $\sum\nolimits_{i = 0}^{N-1}\xi^{i} = 0$. Moreover, for $0\unicode{x2A7D} k\unicode{x2A7D} N-2$, we have $\sum\nolimits_{i = 0}^{k}\xi^{i}\neq 0$. Let *V* be a finite nonempty set and *n* be a nonnegative integer. Let

\begin{align*} C_n = \displaystyle \bigoplus_{v \in V^{n+1}} \mathbb{C}\end{align*}

be the vector space generated by the elements of $V^{n+1}$. For $v = (v_0,v_1\cdots,v_n)\in V^{n+1}$, and $i\in \{0,1,\cdots, n\}$, let $v^{(i)}$ be the sequence obtained from *v* by excluding the *i*th element in the sequence. Define the $(n+1)$th boundary $\mathbb{C}$-linear map $d_{n+1}:C_{n+1}\longrightarrow C_n$ as

\begin{align*} d_{n+1}v = \sum\limits_{i = 0}^{n+1}\xi^{i}v^{\left(i\right)}\end{align*}

and $d_{0} = 0$. For $0\unicode{x2A7D} j_1 < j_2 < \cdots < j_r \unicode{x2A7D} n$, let $v^{(j_1,j_2,\cdots, j_r)}$ denote the sequence obtained from *v* by dropping the elements at positions $j_1,\cdots, j_r$. Then, notice that for $0\unicode{x2A7D} i\unicode{x2A7D} n$, and $0\unicode{x2A7D} j\unicode{x2A7D} n-1$,

\begin{align*}\left(v^{\left(i\right)}\right)^{\left(j\right)} = \left\{ \begin{array}{ll} v^{\left(i,j\right)}, &amp; j < i; \\ v^{\left(i,j+1\right)}, &amp; j\unicode{x2A7E} i. \end{array} \right.\end{align*}

Therefore,

\begin{align*} \begin{split} d_n^2v = d_{n-1}d_nv &amp; = \sum_{i = 0}^n\sum_{j = 0}^{n-1} \xi^i\xi^j\left(v^{\left(i\right)}\right)^{\left(j\right)} = \sum_{i = 0}^n \left(\sum_{0\unicode{x2A7D} j < i} \xi^i\xi^jv^{\left(i, j\right)}+ \sum_{i\unicode{x2A7D} j < n-1} \xi^i\xi^j v^{\left(i,j+1\right)} \right)\\ &amp; = \left(1+\xi\right)\sum_{0\unicode{x2A7D} i < j\unicode{x2A7D} n} \xi^{i+j-1}v^{\left(i,j\right)}. \end{split}\end{align*}

Through induction on $2\unicode{x2A7D} r\unicode{x2A7D} N$, we establish that

\begin{align*} d_n^{r}v = \left(\prod\limits_{k = 1}^{r}\left(1+\xi+\cdots+\xi^{k-1}\right)\right)\sum\limits_{0\unicode{x2A7D} j_{1} < \cdots < j_{r}\unicode{x2A7D} n}\xi^{j_{1}+\cdots+j_{r}-\frac{r\left(r-1\right)}{2}}v^{\left(j_1,j_2,\cdots, j_r\right)}.\end{align*}

Here, $v^{(j_1,j_2,\cdots, j_r)}$ denotes the sequence obtained from *v* by excluding the elements at positions $j_1,\cdots, j_r$. Consequently, based on previous observations, we deduce that $d_n^{N} = 0$. Thus, $(C_*,d)$ forms an *N*-chain complex.

Given $\sigma \in V^{n+1}$ and $ \tau \in V^{n-q+1}$, equation (6) provides an explicit formula for computing the representation matrix $\mathbf{B}_{n,q}$ for $1\unicode{x2A7D} q \unicode{x2A7D} N-1, n\unicode{x2A7E} 1$. Let $\alpha_q = \xi^{-\frac{q(q-1)}{2}}\prod\nolimits_{k = 1}^{q}(1+\xi+\cdots+\xi^{k-1})$, and observe that

**Figure 1.**
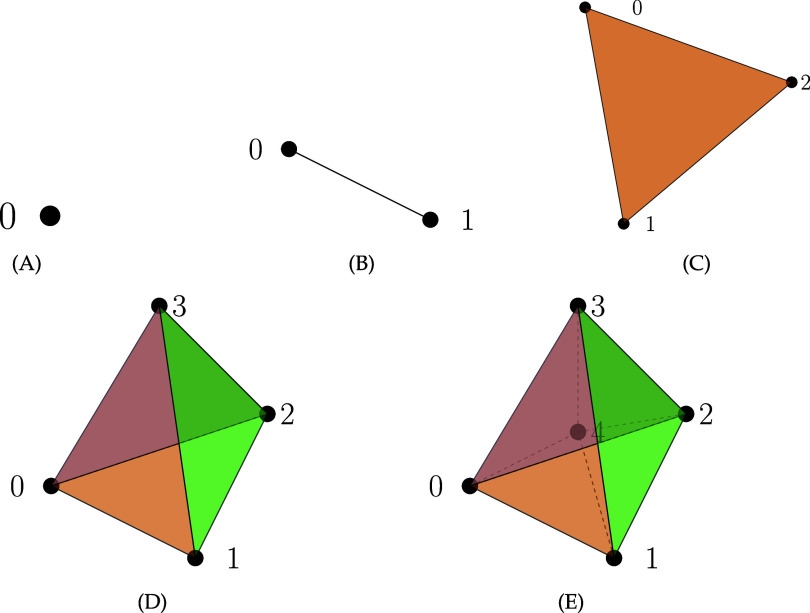
(A) 0-dimensional simplex *σ*[0], a point. (B) 1-dimensional simplex *σ*[1], a line. (C) 2-dimensional simplex *σ*[2], a triangle. (D) 3-dimensional simplex *σ*[3], a tetrahedron.(E) A representation of the boundaries (faces) of a 4-dimensional simplex *σ*[4], a 5-cell.

\begin{align*} \mathbf{B}_{n,q}\left(\tau,\sigma\right) = \alpha_q\sum\limits_{\substack{|j| = q \\ \tau = \sigma ^{j}}}\xi^{\left[j\right]}\end{align*}

where $j = (j_{1}, \cdots, j_{q})$ with $0\unicode{x2A7D} j_{1} < \cdots < j_{q}\unicode{x2A7D} n$, $[j] = j_1+\cdots j_q$, and *σ*^*j*^ denotes the sequence obtained by removing the elements at positions $j_1,\cdots, j_q$. This formula is applicable for deriving explicit expressions for Laplacian and Dirac operators. In simplicial complexes and simplices such as the ones depicted in figure 1, *σ*^*j*^ denotes the simplex obtained from *σ* by excluding the vertices at positions $j_1,\cdots,j_q$. For $n\unicode{x2A7E} 0$, we denote a simplex of dimension *n* by $\sigma[n]$. Table 1 presents the non-zero Mayer Betti numbers of the 3-chain and 5-chain complexes associated with the first seven simplices.

**Table 1.** A table of none-zero Mayer Betti numbers of the first seven simplices for $N = 3, 5$. The table suggests that each simplex may have unique Mayer Betti numbers.

$\sigma[n]$ *N*	*N* = 3	*N* = 5
$\sigma[0]$	$\beta_{0,1} = 1$, $\beta_{0,2} = 1$	$\beta_{0,1} = \beta_{0,2} = \beta_{0,3} = \beta_{0,4} = 1$

$\sigma[1]$	$\beta_{0,1} = 2$, $\beta_{0,2} = 1$, $\beta_{1,2} = 1$	$\beta_{0,1} = \beta_{0,2} = \beta_{0,3} = 2$,
		$\beta_{0,4} = \beta_{1,2} = \beta_{1,3} = \beta_{1,4} = 1$

$\sigma[2]$	$\beta_{0,1} = 2$, $\beta_{1,2} = 2$	$\beta_{0,1} = \beta_{0,2} = 3$, $\beta_{0,3} = 2$,
		$\beta_{1,2} = \beta_{1,3} = 3$, $\beta_{1,4} = 2$,
		$\beta_{2,3} = \beta_{2,4} = 1$

$\sigma[3]$	$\beta_{0,1} = 1$, $\beta_{1,1} = 1$, $\beta_{1,2} = 2$	$\beta_{0,1} = 4$, $\beta_{0,2} = 3$, $\beta_{1,1} = 2$,
		$\beta_{1,2} = 6$, $\beta_{1,3} = 5$, $\beta_{1,4} = 2$,
		$\beta_{2,3} = 4$, $\beta_{2,4} = 3$, $\beta_{3,4} = 1$

$\sigma[4]$	$\beta_{0,1} = 1$, $\beta_{1,1} = 1$, $\beta_{1,2} = 1$, $\beta_{2,2} = 1$	$\beta_{0,1} = 4$, $\beta_{1,1} = 5$, $\beta_{1,2} = 9$,
		$\beta_{2,2} = 5$, $\beta_{2,3} = 9$, $\beta_{2,4} = 5$, $\beta_{3,4} = 4$

$\sigma[5]$	$\beta_{0,1} = 1$, $\beta_{1,2} = 1$, $\beta_{2,1} = 1$, $\beta_{2,2} = 1$	$\beta_{0,1} = 1$, $\beta_{1,1} = 8$, $\beta_{1,2} = 9$, $\beta_{2,2} = 13$,
		$\beta_{2,3} = 14$, $\beta_{2,4} = 5$, $\beta_{3,3} = 8$, $\beta_{3,4} = 9$

$\sigma[6]$	$\beta_{0,1} = 1$, $\beta_{1,2} = 1$, $\beta_{2,1} = 1$, $\beta_{3,2} = 1$	$\beta_{0,1} = 1$, $\beta_{1,1} = 8$, $\beta_{1,2} = 1$, $\beta_{2,1} = 13$,
		$\beta_{2,2} = 21$, $\beta_{2,3} = 14$, $\beta_{3,2} = 13$, $\beta_{3,3} = 21$,
		$\beta_{3,4} = 14$

### 2.2. Important families of 2-chains from *N*-chains

Let $(C_*,d)$ be an *N*-chain, and $1\unicode{x2A7D} q\unicode{x2A7D} N-1$. Then, in the definitions of Mayer homology groups, we considered the sequences of the form

\begin{align*} C_{n+N-q} \xrightarrow[~~~~~~~~~~~~~~]{d_{n+N-q}^{N-q}} C_{n} \xrightarrow[~~~~~~~~~~~~~~]{d_{n}^{q}} C_{n-q}.\end{align*}

Let $0\unicode{x2A7D} i < N-q$, and define the chain complex $(\Omega_{*, i , q}, \partial_{*, i,q})$ to be the chain complex

\begin{align*} \cdots \cdots \xrightarrow[]{~~~~~~} C_{i+2N} \xrightarrow[~~~~~~]{d_{i+2N}^{N-q}} C_{i+N+q} \xrightarrow[~~~~~~]{d_{i+N+q}^{q}} C_{i+N} \xrightarrow[~~~~~~]{d_{i+N}^{N-q}} C_{i+q} \xrightarrow[~~~~~~]{d_{i+q}^q} C_{i} \xrightarrow[~~~~~~]{0} 0,\end{align*}

where

\begin{align*}\Omega_{j, i,q} = \left\{ \begin{array}{ll} C_{i+\frac{j-1}{2}N+q}, &amp; j\ \hbox{is odd;} \\ C_{i+\frac{j}{2}N}, &amp; j\ \hbox{is even,} \end{array} \right.\end{align*}

and

\begin{align*}\mathbf{\partial}_{j, i,q} = \left\{ \begin{array}{ll} d_{i+\frac{j-1}{2}N+q}^{q}, &amp; j\ \hbox{is odd;} \\ d_{i+\frac{j}{2}N}^{N-q}, &amp; j\ \hbox{is positive and even;}\\ 0, &amp; j = 0. \end{array} \right.\end{align*}

Let $\mathbf{T}_{j,i,q}$ denote the representation matrix of $\partial_{j,i,q}$, then

\begin{align*}\mathbf{T}_{j, i,q} = \left\{ \begin{array}{ll} \mathbf{B}_{i+\frac{j-1}{2}N+q,q}, &amp; j\ \hbox{is odd;} \\ \mathbf{B}_{i+\frac{j}{2}N,N-q}, &amp; j\ \hbox{is positive and even;}\\ 0, &amp; j = 0. \end{array} \right.\end{align*}

One advantage of these sequences is that every Mayer’s homology group appears as a homology group of exactly one pair of (*i*, *q*). Precisely,

\begin{align*}H_{j, i,q}\left(\Omega_{*,i,q}\right) = \left\{ \begin{array}{ll} H_{i+\frac{j-1}{2}N+q,q}\left(C_*\right), &amp; j\ \hbox{is odd;} \\ H_{i+\frac{j}{2}N,N-q}\left(C_*\right), &amp; j\ \hbox{is even.} \end{array} \right.\end{align*}

A second advantage is that they will be used in defining Mayer Dirac operators.

**Figure 2.**
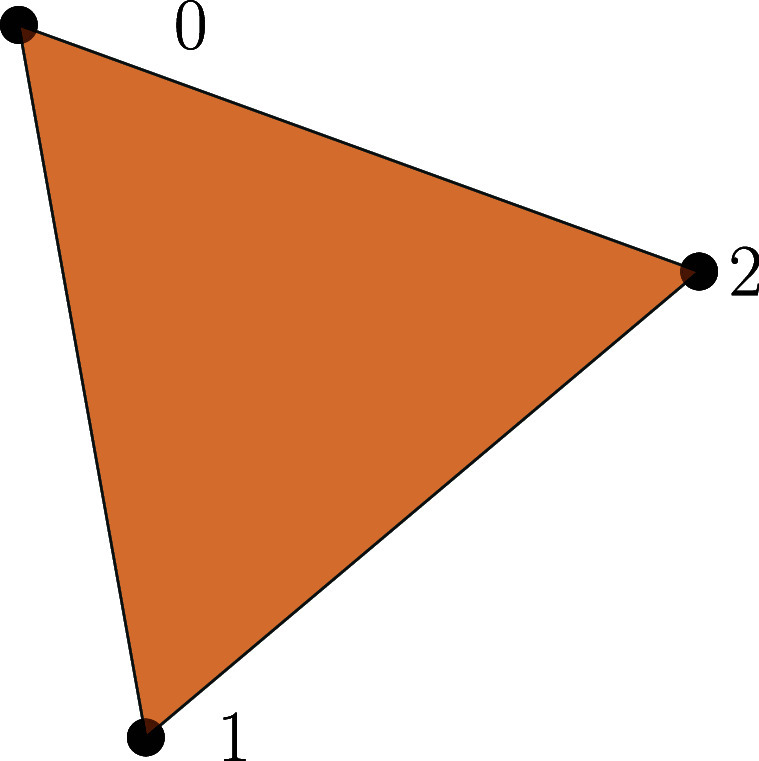
A 2-dimensional simplex *σ*[2], i.e., a triangle.

Example 3. Consider the 2-dimensional simplex depicted in figure 2, $\sigma[2]$, with faces

\begin{align*} \begin{split} &amp; \left(0\right), \left(1\right), \left(2\right), \\ &amp; \left(0,1\right), \left(0,2\right), \left(1,2\right),\\ &amp;\left(0,1,2\right). \end{split}\end{align*}

Consider the 3-chain complex $(C_{\ast}, d)$ induced by $\sigma[2]$ with the boundary operators given by

\begin{align*} \begin{split} d_{2}\left(0,1,2\right)&amp; = \left(1,2\right)+\xi\left(0,2\right)+\xi^{2}\left(0,1\right),\\ d_{1}\left(0, 1\right)&amp; = \left(1\right)+\xi \left(0\right),\\ d_{1}\left(0, 2\right)&amp; = \left(2\right)+\xi \left(0\right),\\ d_{1}\left(1,2\right)&amp; = \left(2\right)+\xi \left(1\right) .\\ \end{split}\end{align*}

The representation matrices of *d*_1_ and *d*_2_ with the simplices as basis are given by

\begin{align*} \mathbf{B}_{1} = \left( \begin{array}{cccc} \xi &amp; \xi &amp; 0 \\ 1 &amp; 0 &amp; \xi \\ 0 &amp; 1 &amp; 1 \end{array} \right),\quad \mathbf{B}_{2} = \left( \begin{array}{c} \xi^{2}\\ \xi \\ 1 \end{array} \right).\end{align*}

The representation matrix of $d_{1}d_{2}$ is

\begin{align*} \mathbf{B}_{1}\mathbf{B}_{2} = \left( \begin{array}{cccc} \xi^2 + \xi^3 \\ \xi+\xi^2 \\ 1+\xi \end{array} \right).\end{align*}

The families of 2-chains are

\begin{align*} 0 \xrightarrow[]{~~~~~~} C_{1} \xrightarrow[]{~~~~~~} C_{0} \xrightarrow[]{~~~~~~} 0,\end{align*}

\begin{align*} 0 \xrightarrow[]{~~~~~~} C_{2} \xrightarrow[]{~~~~~~} C_{0} \xrightarrow[]{~~~~~~} 0,\end{align*}

\begin{align*} 0 \xrightarrow[]{~~~~~~} C_{2} \xrightarrow[]{~~~~~~} C_{1} \xrightarrow[]{~~~~~~} 0.\end{align*}

A straightforward calculation shows that

\begin{align*} \begin{split} Z_{0,1} &amp; = Z_{0,2} = \mathrm{span}\left(\left\{\left(0\right), \left(1\right), \left(2\right)\right\}\right), \\ Z_{1,2} &amp; = \mathrm{span}\left(\left\{\left(0,1\right), \left(0,2\right), \left(1,2\right)\right\}\right), \\ Z_{n,q} &amp; = 0\,\,\hbox{otherwise.} \end{split}\end{align*}

Furthermore,

\begin{align*} \begin{split} \dim\left(B_{0,1}\right)&amp; = {\mathrm{rank}\hspace{0.1em}}\left(\mathbf{B}_1\mathbf{B}_2\right) = 1, \\ \dim\left(B_{0,2}\right)&amp; = {\mathrm{rank}\hspace{0.1em}}\left(\mathbf{B}_1\right) = 3,\\ \dim\left(B_{1,2}\right)&amp; = {\mathrm{rank}\hspace{0.1em}}\left(\mathbf{B}_2\right) = 1, \\ \dim\left(B_{n,q}\right) &amp; = 0\,\,\hbox{otherwise.} \end{split}\end{align*}

By definition, Mayer homology groups are then given by

\begin{align*} \begin{split} H_{0,1}&amp;\cong H_{1,2}\cong \mathbb{C}^2, \\ H_{n,q}&amp; = 0\,\,\hbox{otherwise.} \end{split}\end{align*}

However, the simplicial homology of $\sigma[2]$ is

\begin{align*}H_{n} = \left\{ \begin{array}{ll} \mathbb{C}, &amp; n = 0; \\ 0, &amp; \mathrm{otherwise.} \end{array} \right.\end{align*}

## 3. Dirac operators of *N*-chain complexes

As the constructed *N*-chain complex in example 2, $(C_*,d)$, is over the field $\mathbb{C}$, we introduce the following inner product:

\begin{align*} \langle \lambda\sigma,\mu\tau\rangle = \left\{ \begin{array}{ll} \lambda\cdot\overline{\mu}\cdot w_{\sigma}^2, &amp; \sigma = \tau; \\ 0, &amp; \mathrm{otherwise} \end{array} \right.\end{align*}

for $n\unicode{x2A7E} 0$, $\lambda,\mu\in \mathbb{C}$, and $\sigma, \tau \in V^{n+1}$. The symbol $\overline{\mu}$ represents the complex conjugate of *µ*, and $w_{\sigma} > 0$ denotes a selected weight that can incorporate additional information about the *N*-chain. The notation $w_\sigma^2$ is employed for simplicity. In the case where $w_{\sigma} = 1$ for all $\sigma \in V^{n+1},\ n\unicode{x2A7E} 0$, the *N*-chain is termed unweighted; otherwise, it is referred to as weighted. This inner product will define Laplacians and Diracs on the *N*-chain complexes examined in this work. For a simplicial complex *K* with a set of vertices *V*, and $1\unicode{x2A7D} q\unicode{x2A7D} N-1$, we define the *N*-chain complex on *K* as the *Mayer homology of* *K*, consisting of the *N*-subchain generated by sequences $(v_0,\cdots,v_n)$ such that $v_i < v_j$ in accordance with the partial order of *K*, for *i* < *j*.

### 3.1. Laplacians and Diracs of 2-chain complexes

Let $(C_*, d)$ be a chain complex consisting of vector spaces over $\mathbb{K}$. Then, for a fixed basis for each *C_n_* equipped with the standard inner product, e.g. the basis consists of orthonormal elements, the cochain $(C_*, d^*)$ is then defined to be $d^*_n: C_{n-1} \to C_n$ for $n\unicode{x2A7E} 1$, and $d^*_0: 0 \to C_0$ is the zero map. Recall that $d,\ d^*: \widehat{C}_* \to \widehat{C}_*$. The *n*th Laplacian operator $\Delta_n: C_n\to C_n$ is defined to be

\begin{align*} \Delta_n = \Delta_n^\textrm{Up}+\Delta_n^\textrm{Down},\end{align*}

where $\Delta_n^\textrm{Up} = d_{n+1}d_{n+1}^*$, $\Delta_n^\textrm{Down} = d^*_nd_n$, and $d^*_n$ is the adjoint operator of *d_n_*.

Laplacian operators possess several noteworthy algebraic characteristics with significant computational implications. Initially, Laplacian operators exhibit the properties of being Hermitian, e.g. self-adjoint. That is, for all $n\unicode{x2A7E}0$, and $x,y\in C_n$, we have

\begin{align*} \langle x, \Delta_n y \rangle = \langle \Delta_n x, y \rangle.\end{align*}

Consequently, all their eigenvalues are real. Moreover,

\begin{align*} \langle x, \Delta_n x \rangle \unicode{x2A7E} 0.\end{align*}

Moreover, therefore, $\Delta_n$ is a positive semi-definite operator. Hence, all of their eigenvalues are non-negative. Furthermore, Laplacian operators demonstrate a range of decompositional and homological properties that hold considerable computational importance. For instance, for $n\unicode{x2A7E} 0$,

\begin{align*}C_{n} = \ker\left( \Delta_{n}\right)\oplus \mathrm{Im}d_{n+1}\oplus\mathrm{Im}\left(d_{n}^{\ast}\right),\end{align*}

and

\begin{align*} \ker \Delta_{n} = \ker \left(d_{n}\right)\cap \ker \left(d_{n+1}^{\ast}\right)\cong H_{n}\left(C_*\right).\end{align*}

Therefore, the Betti numbers *β*_*n*_ are given by

\begin{align*} \beta_n = \dim\left(H_{n}\left(C_*\right)\right) = \dim\left(\ker \Delta_{n}\right) = \eta\left(\Delta_n\right)\end{align*}

where for a linear operator *T* between two finite dimensional vector spaces, $\eta(T)$ denotes the nullity of *T*. We recommend referring to [35] for details. An essential observation of computational importance is that given an orthonormal basis $\{v_i\}$ of *C_n_*, the matrix representation $\mathbf{L}^\textrm{Down}_n$ of $\Delta^\textrm{Down}_n$ can be given by

\begin{align*} \mathbf{L}^\textrm{Down}_n\left(v_i,v_j\right) = \langle d_n\left(v_j\right), d_n\left(v_i\right) \rangle,\end{align*}

and the matrix representation $\mathbf{L}^\textrm{Up}_n$ of $\Delta^\textrm{Up}_n$ can be given by

\begin{align*} \mathbf{L}^\textrm{Up}_n\left(v_i,v_j\right) = \langle d^*_{n+1}\left(v_j\right), d^*_{n+1}\left(v_i\right) \rangle.\end{align*}

The Laplacian operator $\Delta: \widehat{C}_* \to \widehat{C}_*$ is given by $\Delta = d^*d+dd^*$, and it satisfies

**Figure:**
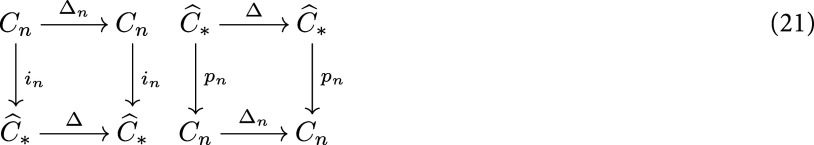


where *i_n_* is the inclusion map, and *p_n_* is the projection map.

For an integer $k\unicode{x2A7E} 0$, the *k*th Dirac operator *D_k_* is defined to be the operator with the matrix representation given by

\begin{align*} \mathbf{D}_k = \begin{pmatrix} \textbf{0}_{n_0\times n_0} &amp; \mathbf{B}_1 &amp; \textbf{0}_{n_0\times n_2} &amp; \cdots &amp; \textbf{0}_{n_0\times n_{k}} &amp; \textbf{0}_{n_0\times n_{k+1}} \\ \mathbf{B}_1^* &amp; \textbf{0}_{n_1\times n_1} &amp; \mathbf{B}_2 &amp; \cdots &amp; \textbf{0}_{n_1\times n_{k}} &amp; \textbf{0}_{n_1\times n_{k+1}} \\ \textbf{0}_{n_2\times n_0} &amp; \mathbf{B}_2^* &amp; \textbf{0}_{n_2\times n_2} &amp; \cdots &amp; \textbf{0}_{n_2\times n_{k}} &amp; \textbf{0}_{n_2\times n_{k+1}} \\ \vdots &amp; \vdots &amp; \vdots &amp; \ddots &amp; \vdots &amp; \vdots \\ \textbf{0}_{n_{k}\times n_0} &amp; \textbf{0}_{n_{k}\times n_1} &amp; \textbf{0}_{n_{k}\times n_2} &amp; \cdots &amp; \textbf{0}_{n_{k} \times n_{k}} &amp; \mathbf{B}_{k+1}\\ \textbf{0}_{n_{k+1}\times n_0} &amp; \textbf{0}_{n_{k+1}\times n_1} &amp; \textbf{0}_{n_{k+1}\times n_2} &amp; \cdots &amp; \mathbf{B}_{k+1}^* &amp; \textbf{0}_{n_{k+1}\times n_{k+1}} \end{pmatrix},\end{align*}

where **B**_*n*_ is the matrix representation of *d_n_*, and $\mathbf{B}_n^*$ is its adjoint matrix.

Similar to Laplacian operators, Dirac operators exhibit numerous algebraic properties and possess certain connections with Laplacian operators, as discussed in [35]. Firstly, for a non-negative integer *k*, the operator *D_k_* is Hermitian and self-adjoint; consequently, all its eigenvalues are real. Furthermore, its spectrum is symmetric; *λ* is an eigenvalue of *D_k_* if and only if $-\lambda$ is. Secondly,

\begin{align*} \mathbf{D}_k^2 = \begin{pmatrix} \mathbf{L}_0 &amp; \textbf{0}_{n_0\times n_1} &amp; \textbf{0}_{n_0\times n_2} &amp; \cdots &amp; \textbf{0}_{n_0\times n_k} &amp; \textbf{0}_{n_0\times n_{k+1}} \\ \textbf{0}_{n_1\times n_0} &amp; \mathbf{L}_1 &amp; \textbf{0}_{n_1\times n_2} &amp; \cdots &amp; \textbf{0}_{n_1\times n_k} &amp; \textbf{0}_{n_1\times n_{k+1}} \\ \textbf{0}_{n_2\times n_{0}} &amp; \textbf{0}_{n_2\times n_{1}} &amp; \mathbf{L}_2 &amp; \cdots &amp; \textbf{0}_{n_2\times n_{k}} &amp; \textbf{0}_{n_2\times n_{k+1}} \\ \vdots &amp; \vdots &amp; \vdots &amp; \ddots &amp; \vdots &amp; \vdots \\ \textbf{0}_{n_{k}\times n_{0}} &amp; \textbf{0}_{n_{k}\times n_{1}} &amp; \textbf{0}_{n_{k}\times n_{2}} &amp; \cdots &amp; \mathbf{L}_k &amp; \textbf{0}_{n_{k}\times n_{k+1}} \\ \textbf{0}_{n_{k+1}\times n_{0}} &amp; \textbf{0}_{n_{k+1}\times n_{1}} &amp;\textbf{0}_{n_{k+1}\times n_{2}} &amp; \cdots &amp; \textbf{0}_{n_{k+1}\times n_{k}} &amp; \mathbf{L}^\textrm{Down}_{k+1} \end{pmatrix},\end{align*}

where **L**_*n*_ is the matrix representation of $\Delta_n$, and $\mathbf{L}^\textrm{Down}_{k+1}$ is the representation matrix for $\Delta_{k+1}^\textrm{Down}$. Therefore,

\begin{align*} \textrm{ker}\left(D_k\right) = \textrm{ker}\left(D_k^2\right)\cong\left(\bigoplus_{i = 0}^{k}\textrm{ker}\left(\Delta_i\right)\right)\bigoplus\textrm{ker}\left(\Delta_{k+1}^\textrm{Down}\right)\end{align*}

and

\begin{align*} \eta\left(D_k\right) = \eta\left(\Delta_{k+1}^\textrm{Down}\right)+\sum_{i = 0}^{k}\eta\left(\Delta_i\right),\end{align*}

which implies that $\eta(D_k)\unicode{x2A7E} \sum_{i = 0}^{k}\eta(\Delta_i)$ with equality if and only if $ \eta(\Delta_{k+1}^\textrm{Down}) = 0$, that is, if and only if $d_{k+1}$ is injective.

Notice that $D_k : \bigoplus_{n = 0}^{k+1} C_{n} \to \bigoplus_{n = 0}^{k+1} C_{n}$. We define the Dirac operator $D : \widehat{C}_* \to \widehat{C}_*$ to be given by $D = d+d^*$. The restriction of *D* to every $\bigoplus_{n = 0}^{k} C_{n}$ is equal to *D_k_*. Therefore, $D^2 = \Delta.$

### 3.2. The Mayer Diracs on *N*-chain complexes

Let $(C_*,d)$ be an *N*-chain. For $1\unicode{x2A7D} q\unicode{x2A7D} N-1$ and $n\unicode{x2A7E} 0$, the *q*th *Mayer Laplacian* at *n* is defined as the Laplacian at *C_n_* in the sequence

\begin{align*} C_{n+N-q} \xrightarrow[~~~~~~~~~~~~~]{d_{n+N-q}^{N-q}} C_{n} \xrightarrow[~~~~~~~~~~~~~~]{d_{n}^{q}} C_{n-q}.\end{align*}

and is given by

\begin{align*} \Delta_{n,q}: = d_{n+N-q}^{N-q}\circ \left(d_{n+N-q}^{N-q}\right)^*+ \left(d_{n}^{q}\right)^*\circ d_{n}^{q}.\end{align*}

Then the representation matrix of $\Delta_{n,q}$ is given by

\begin{align*} \mathbf{L}_{n,q} = \mathbf{B}_{n+N-q, N-q}\left(\overline{\mathbf{B}}_{n+N-q, N-q}\right)^{T} + \left(\overline{\mathbf{B}}_{n,q}\right)^{T}\mathbf{B}_{n,q}.\end{align*}

where, $\overline{\mathbf{B}}^{T}$ is the conjugate transpose or Hermitian transpose matrix of a matrix **B**, and $\mathbf{B}_{n, q}$ is the representation matrix of $d_{n}^{q}$ with respect an orthonormal basis. Recall that $\mathbf{B}_{n,q}$ is given by the product

\begin{align*} \mathbf{B}_{n, q} = \mathbf{B}_{n-\left(q-1\right)}\cdots \mathbf{B}_{n}\end{align*}

where **B**_*i*_ is the representation matrix of *d*_*i*_ with respect to the chosen orthonormal basis. Per the previous section discussions, the Laplacian $\Delta_{n,q}$ on *C*_*n*_ is a self-adjoint and positive semidefinite operator. Furthermore, for any *n* and $1\unicode{x2A7D} q\unicode{x2A7D} N-1$, we have $\dim\ker\Delta_{n,q} = \beta_{n,q}$, and

\begin{align*}\ker \Delta_{n,q}\cong H_{n,q}.\end{align*}

In the weighted version of the *N*-chain constructed in example 2, the representation matrix of $d_{n}^{q}$ with respect to the orthonormal bases $\{\frac{\sigma}{w_{\sigma}}| \sigma \in V^{n+1}\}$ and $\{\frac{\tau}{w_{\tau}}| \tau \in V^{n-q+1}\}$ is given by

\begin{align*} \mathbf{B}_{n,q}\left(\frac{\tau}{w_{\tau}},\frac{\sigma}{w_{\sigma}}\right) = \sum\limits_{\substack{|j| = q \\ \tau = \sigma ^{j}}} \frac{w_\tau}{w_\sigma}\alpha_q \xi^{\left[j\right]}.\end{align*}

Therefore,

\begin{align*} \mathbf{L}_{n,q}^\textrm{Down}\left(\frac{\tau}{w_{\tau}},\frac{\sigma}{w_{\sigma}}\right) = \sum\limits_{\delta \in V^{n-q+1}}\sum\limits_{\substack{\forall\ j,\ s \\ |j| = |s| = q \\ \tau^{s} = \sigma^{j} = :\delta }} \frac{w^2_\delta|\alpha_q|^2}{w_\sigma w_\tau} \xi^{\left[j\right] -\left[s\right]}.\end{align*}

Furthermore, since for $\tau \in V^{n+N-q+1}$ and $ \sigma \in V^{n+1}$ we have

\begin{align*} \overline{\mathbf{B}}^T_{n+N-q,N-q}\left(\frac{\tau}{w_{\tau}},\frac{\sigma}{w_{\sigma}}\right) = \sum\limits_{\substack{|j| = N-q\\ \sigma = \tau^{j}}} \frac{w_\sigma}{w_\tau}\overline{\alpha}_{N-q} \xi^{-\left[j\right]},\end{align*}

then

\begin{align*} \mathbf{L}_{n,q}^\textrm{Up}\left(\frac{\tau}{w_{\tau}},\frac{\sigma}{w_{\sigma}}\right) = \sum\limits_{\substack{\delta \in V^{n+N-q+1}}}\sum\limits_{\substack{\forall\ j,\ s \\ |j| = |s| = N-q \\ \delta^{s} = \sigma,\ \delta^{j} = \tau }} \frac{w_\tau w_\sigma|\alpha_{N-q}|^2}{w^2_\delta} \xi^{\left[j\right]-\left[s\right]}.\end{align*}

Hence, in the case of simplicial complexes, whenever there are two simplexs such that one of them has more than *q* vertices that are not vertices in the other simplex, then their images under $d_n^q$ are orthogonal. Similarly, if these two simplexes do not have a common simplex of dimension $n+N-q$, such that they are faces of that complex, then their images under $(d_{n+N-q}^{N-q})^*$ are orthogonal. This shows that the Mayer homology and Mayer Laplacian of a complex capture further relations between simplexes of different dimensions. The *q*th Laplacian operator $\widehat{\Delta}_q: \widehat{C}_* \to \widehat{C}_*$ is the linear map such that its restriction to *C_n_* is equal to $\Delta_{n,q}$.

Let $(C_*,d)$ be an *N*-chain, $1\unicode{x2A7D} q \unicode{x2A7D} N-1$, $0\unicode{x2A7D} i < N-q$, and $k\unicode{x2A7E} 0$ be integers. The $(k,i,q)$-Dirac operator of the Mayer *N*-chain complex $(C_*,d)$ is defined to be the *k*th Dirac operator of the special 2-chain complex $\Omega_{*,i,q}$

\begin{align*} \cdots C_{i+2N}\xrightarrow[~~~~~~]{d_{i+2N}^{N-q}} C_{i+N+q}\xrightarrow[~~~~~~]{d_{i+N+q}^{q}} C_{i+N}\xrightarrow[~~~~~~]{d_{i+N}^{N-q}} C_{i+q}\xrightarrow[~~~~~~]{d_{i+q}^{q}} C_{i}\xrightarrow[]{~~~~~~} 0\end{align*}

whose matrix representation is given by

\begin{align*} \mathbf{D}_{k, i,q} = \begin{pmatrix} \textbf{0}_{n_0\times n_0} &amp; \mathbf{T}_{1, i,q} &amp; \textbf{0}_{n_0\times n_2} &amp; \cdots &amp; \textbf{0}_{n_0\times n_{k}} &amp; \textbf{0}_{n_0\times n_{k+1}} \\ \mathbf{T}_{1, i,q}^* &amp; \textbf{0}_{n_1\times n_1} &amp; \mathbf{T}_{2, i,q} &amp; \cdots &amp; \textbf{0}_{n_1\times n_{k}} &amp; \textbf{0}_{n_1\times n_{k+1}} \\ \textbf{0}_{n_2\times n_0} &amp; \mathbf{T}_{2, i,q}^* &amp; \textbf{0}_{n_2\times n_2} &amp; \cdots &amp; \textbf{0}_{n_2\times n_{k}} &amp; \textbf{0}_{n_2\times n_{k+1}} \\ \vdots &amp; \vdots &amp; \vdots &amp; \ddots &amp; \vdots &amp; \vdots \\ \textbf{0}_{n_{k}\times n_0} &amp; \textbf{0}_{n_{k}\times n_1} &amp; \textbf{0}_{n_{k}\times n_2} &amp; \cdots &amp; \textbf{0}_{n_{k} \times n_{k}} &amp; \mathbf{T}_{k+1, i,q}\\ \textbf{0}_{n_{k+1}\times n_0} &amp; \textbf{0}_{n_{k+1}\times n_1} &amp; \textbf{0}_{n_{k+1}\times n_2} &amp; \cdots &amp; \mathbf{T}_{k+1, i,q}^* &amp; \textbf{0}_{n_{k+1}\times n_{k+1}} \end{pmatrix}.\end{align*}

In this case, we observe that

\begin{align*} \mathbf{D}_{k, i,q}^2 = \begin{pmatrix} \widehat{\mathbf{L}}_{0, i,q} &amp; \textbf{0}_{n_0\times n_1} &amp; \textbf{0}_{n_0\times n_2} &amp; \cdots &amp; \textbf{0}_{n_0\times n_k} &amp; \textbf{0}_{n_0\times n_{k+1}} \\ \textbf{0}_{n_1\times n_0} &amp; \widehat{\mathbf{L}}_{1, i,q} &amp; \textbf{0}_{n_1\times n_2} &amp; \cdots &amp; \textbf{0}_{n_1\times n_k} &amp; \textbf{0}_{n_1\times n_{k+1}} \\ \textbf{0}_{n_2\times n_{0}} &amp; \textbf{0}_{n_2\times n_{1}} &amp; \widehat{\mathbf{L}}_{2, i,q} &amp; \cdots &amp; \textbf{0}_{n_2\times n_{k}} &amp; \textbf{0}_{n_2\times n_{k+1}} \\ \vdots &amp; \vdots &amp; \vdots &amp; \ddots &amp; \vdots &amp; \vdots \\ \textbf{0}_{n_{k}\times n_{0}} &amp; \textbf{0}_{n_{k}\times n_{1}} &amp; \textbf{0}_{n_{k}\times n_{2}} &amp; \cdots &amp; \widehat{\mathbf{L}}_{k, i,q} &amp; \textbf{0}_{n_{k}\times n_{k+1}} \\ \textbf{0}_{n_{k+1}\times n_{0}} &amp; \textbf{0}_{n_{k+1}\times n_{1}} &amp;\textbf{0}_{n_{k+1}\times n_{2}} &amp; \cdots &amp; \textbf{0}_{n_{k+1}\times n_{k}} &amp; \widehat{\mathbf{L}}^\textrm{Down}_{k+1, i,q} \end{pmatrix},\end{align*}

where

\begin{align*}\widehat{\mathbf{L}}_{j, i,q} = \left\{ \begin{array}{ll} \mathbf{L}_{i+\frac{j-1}{2}N+q,q}, &amp; j\ \hbox{is odd;} \\ \mathbf{L}_{i+\frac{j}{2}N,N-q}, &amp; j\ \hbox{is even.} \end{array} \right.\end{align*}

According to previous discussions, Mayer Dirac operators are Hermitian and self-adjoint; consequently, all their eigenvalues are real, and their spectrum is symmetric as well. Furthermore, $D_{k,i,q}$ captures cumulative information of alternating sequence of Mayer Laplacians of the form $L_{t,q}$ and $L_{t,N-q}$ in addition to $L_{t,q}^\textrm{Down}$. Therefore,

\begin{align*} \begin{split} \textrm{ker}\left(D_{k, i,q}\right) = \textrm{ker}\left(D_{k, i,q}^2\right)&amp;\cong\left(\bigoplus_{j = 0}^{k}\textrm{ker}\left(\widehat{\mathbf{L}}_{j, i,q}\right)\right)\bigoplus\textrm{ker}\left(\widehat{\mathbf{L}}_{k+1, i, q}^\textrm{Down}\right) \\ &amp;\cong\left(\bigoplus_{j\ \textrm{odd}}\textrm{ker}\left(\mathbf{L}_{t_j,q}\right)\right)\bigoplus\left(\bigoplus_{j \ \textrm{even}}\textrm{ker}\left(\mathbf{L}_{t_j,N-q}\right)\right)\bigoplus\textrm{ker}\left(\widehat{\mathbf{L}}^\textrm{Down}_{k+1, i,q}\right) \end{split}\end{align*}

and

\begin{align*} \eta\left(D_{k,i,q}\right) = \eta\left(\widehat{\mathbf{L}}_{k+1, i , q}^\textrm{Down}\right)+\sum_{j \ \textrm{odd}}\eta\left(\mathbf{L}_{t_j,q}\right)+\sum_{j \ \textrm{even}}\eta\left(\mathbf{L}_{t_j,N-q}\right),\end{align*}

where

\begin{align*}t_j = \left\{ \begin{array}{ll} i+\frac{j-1}{2}N+q, &amp; j\ \hbox{is odd;} \\ i+\frac{j}{2}N, &amp; j\ \hbox{is even.} \end{array} \right.\end{align*}

As in the case of 2-chains, the (*i*, *q*)-Dirac operator $D_{i,q}$ on $\widehat{\Omega}_{*,i,q}$ is defined as the linear map whose restriction on each $\bigoplus_{n = 0}^{k}\Omega_{n,i,q}$ is $D_{k,i,q}$. Since each $L_{t,q}$ appears as a Laplacian in $\widehat{\Omega}_{*,i,q}$ for a unique pair (*i*, *q*) such that $1\unicode{x2A7D} q\unicode{x2A7D} N-1$ and $0\unicode{x2A7D} i < N-q$, and since $ \bigoplus\nolimits_{i,q}\widehat{\Omega}_{*,i,q} = \bigoplus^{N-1}_{1} \widehat{C}_*$, then with suitable labeling and identification, we obtain

\begin{align*} \left(\bigoplus_{i,q} D_{i,q}\right)^2 = \bigoplus_q \widehat{\Delta}_q.\end{align*}

When *N* = 2, there is only $\widehat{\Delta}_1 = \Delta$, and $D_{0,1} = D$, and therefore we retreive the classical case.

In the case of simplicial complexes, the formulas of the boundary maps and Laplacians become relatively simple. Given a simplex $\sigma \in K$ of dimension *n* and a face $\tau \subset \sigma$ of dimension *n* − *q*, there exists a unique $j = (j_1,\cdots, j_q)$ with $j_1 < \cdots < j_q$ such that $\sigma^j = \tau$. Therefore,

\begin{align*}\mathbf{B}_{n,q}\left(\frac{\tau}{w_{\tau}},\frac{\sigma}{w_{\sigma}}\right) = \left\{ \begin{array}{ll} \frac{w_\tau}{w_\sigma}\alpha_q \xi^{\left[j\right]}, &amp; \sigma^j = \tau; \\ 0, &amp; \hbox{otherwise.} \end{array} \right.\end{align*}

Hence,

\begin{align*} \mathbf{L}_{n,q}^\textrm{Down}\left(\frac{\tau}{w_{\tau}},\frac{\sigma}{w_{\sigma}}\right) = \sum\limits_{\substack{\forall\ j,\ s \\ |j| = |s| = q \\ \tau^{s} = \sigma^{j} = :\delta }} \frac{w^2_\delta|\alpha_q|^2}{w_\sigma w_\tau} \xi^{\left[j\right] -\left[s\right]}.\end{align*}

Furthermore, given a simplex *τ* of dimension $n+N-q$ and a simplex *σ* of dimension *n*, we have

\begin{align*}\overline{\mathbf{B}}^T_{n+N-q,N-q}\left(\frac{\tau}{w_{\tau}},\frac{\sigma}{w_{\sigma}}\right) = \left\{ \begin{array}{ll} \frac{w_\sigma}{w_\tau}\overline{\alpha}_{N-q} \xi^{-\left[j\right]}, &amp; \tau^j = \sigma; \\ 0, &amp; \hbox{otherwise.} \end{array} \right.\end{align*}

and then

\begin{align*} \mathbf{L}_{n,q}^\textrm{Up}\left(\frac{\tau}{w_{\tau}},\frac{\sigma}{w_{\sigma}}\right) = \sum\limits_{\substack{\forall\ j,\ s \\ |j| = |s| = N-q \\ \delta^{s} = \sigma,\ \delta^{j} = \tau }} \frac{w_\tau w_\sigma|\alpha_{N-q}|^2}{w^2_\delta} \xi^{\left[j\right]-\left[s\right]}.\end{align*}

### Factorization of $\Delta_{q}$

3.3.

Define $d: \widehat{C}\to \widehat{C}$ to be the linear map that sends every element $v \in C_n$ to $d_nv \in C_{n-1}$. Furthermore, define its ‘adjoint’ operator $d^*: \widehat{C}\to \widehat{C}$ to be the linear map that sends every element $v\in C_n$ to $(d^*_n)v \in C_{n+1}$. Moreover, define $(d^q)^*: \widehat{C}\to \widehat{C}$ to be the linear map that sends every $v\in C_n$ to $(d^q_{n+q})^*v$. Notice then that $(d^*)^q = (d^q)^*$. With this setting, we obtain

\begin{align*} \begin{array}{ll} \Delta_{q} &amp; = \left(d^q\right)^*d^q + d^{N-q}\left(d^{N-q}\right)^*\\ &amp; = \left(\left(d^q\right)^* + d^{N-q}\right)\left(d^q + \left(d^{N-q}\right)^*\right) \\ &amp; = X_{N-q}X_{q}, \end{array}\end{align*}

where $X_q = d^q + (d^{N-q})^*$. Inspired by the construction of Dirac operators, we define the operator $X_{k,q}: \bigoplus_{n = 0}^{k+N-q} C_n \to \bigoplus_{n = 0}^{k+N-q} C_n$ to be the linear operator with the block matrix representation given by $\mathbf{X}_{k,q} = (a_{i,j})$ such that

\begin{align*}a_{i,j} = \left\{ \begin{array}{ll} \mathbf{B}_{j, q}, &amp; j = i+q; \\ \left(\mathbf{B}_{i,N- q}\right)^*, &amp; i = j +N-q;\\ 0, &amp; \hbox{otherwise.} \end{array} \right.\end{align*}

where $0\unicode{x2A7D} i,\ j \unicode{x2A7D} k+N-q$. Writing $\mathbf{X}_{k,q}$ in the column representation $\mathbf{X}_{k,q} = (a_0, a_1, \cdots, a_{k+N-q})$, where *a_j_* is a column block, then *a_j_* is given by

\begin{align*} \begin{matrix} \begin{array}{l} ~ \cr C_0 \cr \vdots \cr C_{j+N-q} \cr \vdots \cr C_{k+N-q} \\ \end{array}\quad \begin{array}{ll} \ \ \qquad C_{j:\ j < q}\\ \begin{pmatrix} 0\\ \vdots\\ \left(\mathbf{B}_{j+N-q,N- q}\right)^*\\ \vdots\\ 0\\ \end{pmatrix}, \end{array}\quad \begin{array}{l} ~ \cr C_0 \cr \vdots \cr C_{j-q} \cr \vdots \cr C_{j+N-q} \\ \vdots\\ C_{k+N-q}\\ \end{array}\quad \begin{array}{ll} \qquad C_{j:\ q\unicode{x2A7D} j \unicode{x2A7D} k}\\ \begin{pmatrix} 0\\ \vdots\\ \mathbf{B}_{j, q}\\ \vdots\\ \left(\mathbf{B}_{j+N-q,N- q}\right)^*\\ \vdots\\ 0\\ \end{pmatrix}, \end{array}\quad \begin{array}{l} ~ \cr C_0 \cr \vdots \cr C_{j-q} \cr \vdots \cr C_{k+N-q} \\ \end{array}\quad \begin{array}{ll} C_{j:\ k,\ q < j}\\ \begin{pmatrix} 0\\ \vdots\\ \mathbf{B}_{j, q}\\ \vdots\\ 0\\ \end{pmatrix}. \end{array} \end{matrix}\end{align*}

In this construction it is evident that $\mathbf{X}_{k, N-q} = \mathbf{X}^*_{k, q}$ and

\begin{align*} \mathbf{X}_{k, N-q}\mathbf{X}_{k,q} = \begin{pmatrix} \mathbf{L}_{0, q} &amp; \textbf{0}_{n_0\times n_1} &amp; \cdots &amp; \textbf{0}_{n_0\times n_k} &amp; \textbf{0}_{n_0\times n_{k+1}} &amp; \cdots &amp; \textbf{0}_{n_0\times n_{k+N-q}} \\ \textbf{0}_{n_1\times n_0} &amp; \mathbf{L}_{1, q} &amp; \cdots &amp; \textbf{0}_{n_1\times n_k} &amp; \textbf{0}_{n_1\times n_{k+1}} &amp; \cdots &amp; \textbf{0}_{n_1\times n_{k+N-q}} \\ \vdots &amp; \vdots &amp; \ddots &amp; \vdots &amp; \vdots &amp; \vdots &amp; \vdots \\ \textbf{0}_{n_{k}\times n_{0}} &amp; \textbf{0}_{n_{k}\times n_{1}} &amp; \cdots &amp; \mathbf{L}_{k, q} &amp; \textbf{0}_{n_{k}\times n_{k+1}} &amp; \cdots &amp; \textbf{0}_{n_k\times n_{k+N-q}} \\[4pt] \textbf{0}_{n_{k+1}\times n_{0}} &amp; \textbf{0}_{n_{k+1}\times n_{1}} &amp; \cdots &amp; \textbf{0}_{n_{k+1}\times n_{k}} &amp; \mathbf{L}_{k+1, q}^\textrm{Down} &amp; \cdots &amp; \textbf{0}_{n_{k+1}\times n_{k+N-q}}\\ \vdots &amp; \vdots &amp; \vdots &amp; \vdots &amp; \vdots &amp; \ddots &amp; \vdots \\ \textbf{0}_{n_{k+N-q}\times n_{0}} &amp; \textbf{0}_{n_{k+N-q}\times n_{1}} &amp; \cdots &amp; \textbf{0}_{n_{k+N-q}\times n_{k}} &amp; \textbf{0}_{n_{k+N-q}\times n_{k+1}} &amp; \cdots &amp; \mathbf{L}_{k+N-q, q}^\textrm{Down} \end{pmatrix}.\end{align*}

That is,

\begin{align*} \Delta_{q}|_{\bigoplus_{n = 0}^{k} C_n} = \left(X^*_{k,q}X_{k,q}\right)|_{\bigoplus_{n = 0}^{k} C_n}.\end{align*}

We notice that $\mathbf{X}_{k,q}$ is not Hermitian in general; however, when *N* is even and $q = N/2$, then $\mathbf{X}_{k,q}$ is Hermitian. In particular, when *N* = 2, we retrieve the classical result of Dirac and Laplacian operators. Furthermore, most of the classical results in the previous sections hold true. For instance,

\begin{align*} \textrm{ker}\left(X_{k,q}\right) = \textrm{ker}\left(X_{k,N-q}X_{k,q}\right)\cong\left(\bigoplus_{i = 0}^{k}\textrm{ker}\left(\Delta_{i, q}\right)\right)\bigoplus\left(\bigoplus_{j = k+1}^{k+N-q}\textrm{ker}\left(\Delta_{j, q}^\textrm{Down}\right)\right),\end{align*}

and

\begin{align*} \eta\left(X_{k,q}\right) = \eta\left(X_{k,N-q}X_{k,q}\right) = \left(\sum_{i = 0}^{k}\eta\left(\Delta_{i, q}\right)\right)+\left(\sum_{j = k+1}^{k+N-q}\eta\left(\Delta_{j, q}^\textrm{Down}\right)\right).\end{align*}

Since $X_{k,q}$ is not Hermitian in general, its eigenvalues are not necessarily real. To have a glimpse of the shape of these matrices, we list below the first three of the cases when *N* = 3 and *q* = 1.

\begin{equation*} \mathbf{X}_{0,1} = \begin{pmatrix} \textbf{0}_{n_0\times n_0} &amp; \mathbf{B}_{1,1} &amp; \textbf{0}_{n_0\times n_2} \\ \textbf{0}_{n_1\times n_0} &amp; \textbf{0}_{n_1\times n_1} &amp; \mathbf{B}_{2,1} \\ \left(\mathbf{B}_{2,2}\right)^* &amp; \textbf{0}_{n_2\times n_1} &amp; \textbf{0}_{n_2\times n_2} \end{pmatrix}, \quad \mathbf{X}_{1,1} = \begin{pmatrix} \textbf{0}_{n_0\times n_0} &amp; \mathbf{B}_{1,1} &amp; \textbf{0}_{n_0\times n_2} &amp; \textbf{0}_{n_0\times n_3} \\ \textbf{0}_{n_1\times n_0} &amp; \textbf{0}_{n_1\times n_1} &amp; \mathbf{B}_{2,1} &amp; \textbf{0}_{n_1\times n_3} \\ \left(\mathbf{B}_{2,2}\right)^* &amp; \textbf{0}_{n_2\times n_1} &amp; \textbf{0}_{n_2\times n_2} &amp; \mathbf{B}_{3,1} \\ \textbf{0}_{n_3\times n_0} &amp; \left(\mathbf{B}_{3,2}\right)^* &amp; \textbf{0}_{n_3\times n_2} &amp; \textbf{0}_{n_3\times n_3} \\ \end{pmatrix},\end{equation*}

\begin{equation*} \mathbf{X}_{2,1} = \begin{pmatrix} \textbf{0}_{n_0\times n_0} &amp; \mathbf{B}_{1,1} &amp; \textbf{0}_{n_0\times n_2} &amp; \textbf{0}_{n_0\times n_3} &amp; \textbf{0}_{n_0\times n_4} \\ \textbf{0}_{n_1\times n_0} &amp; \textbf{0}_{n_1\times n_1} &amp; \mathbf{B}_{2,1} &amp; \textbf{0}_{n_1\times n_3} &amp; \textbf{0}_{n_1\times n_4} \\ \left(\mathbf{B}_{2,2}\right)^* &amp; \textbf{0}_{n_2\times n_1} &amp; \textbf{0}_{n_2\times n_2} &amp; \mathbf{B}_{3,1} &amp; \textbf{0}_{n_2\times n_4} \\ \textbf{0}_{n_3\times n_0} &amp; \left(\mathbf{B}_{3,2}\right)^* &amp; \textbf{0}_{n_3\times n_2} &amp; \textbf{0}_{n_3\times n_3} &amp;\mathbf{B}_{4,1} \\ \textbf{0}_{n_4\times n_0} &amp; \textbf{0}_{n_4\times n_1} &amp; \left(\mathbf{B}_{4,2}\right)^* &amp; \textbf{0}_{n_4\times n_3} &amp;\textbf{0}_{n_4\times n_4} \\ \end{pmatrix}.\end{equation*}

## 4. Persistent Dirac on *N*-chain complexes

This section explores the persistent Mayer Dirac on *N*-chain complexes. Since Mayer Dirac defined on *N*-chain complex provides information different from the usual simplicial Dirac, investigating Mayer invariants is highly meaningful for our study of the data’s topological characteristics and geometric structure. From now on, the ground field is the complex number field $\mathbb{C}$. Besides, we always consider the case that *N* is a prime number for the sake of simplicity.

### 4.1. Persistent Mayer homology

Recall that a morphism of *N*-chain complexes $\phi : (A_*, d^A) \to (B_*, d^B)$ is a sequence of linear maps $\phi = (\phi_n: A_n \to B_n)$ such that the following diagram commutes.

**Figure:**
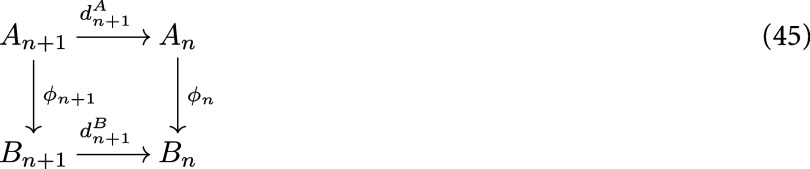


*φ* is called an embedding if each *φ*_*n*_ is injective. A filtration of an *N*-chain complex $(C_*,d)$ is a set of *N*-chain complexes $\{(C_*^{(x)}, d^{(x)})\}_{x\in [a,b]}$ such that $C_*^{(b)} = C_*$, and for $x\unicode{x2A7D} y$, $C_*^{(x)}$ is embedded in $C_*^{(y)}$, and that for $x\unicode{x2A7D} y\unicode{x2A7D} z$ the following diagram commutes.

**Figure:**
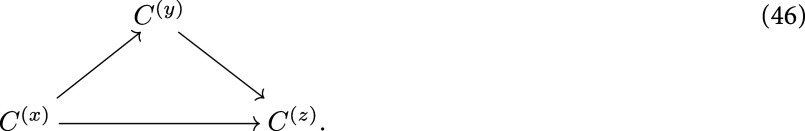


For $1\unicode{x2A7D} q\unicode{x2A7D} N-1, x\unicode{x2A7D} y$, the embedding results in a map

\begin{align*} H_{\ast,q}\left(C^{\left(x\right)}\right)\to H_{\ast,q}\left(C^{\left(y\right)}\right)\end{align*}

and therefore the *q*th *(x, y)-persistent Mayer homology* is defined by

\begin{align*} H_{n,q}^{x,y}: = {\mathrm{im}\hspace{0.1em}} \left(H_{n,q}\left(C^{\left(x\right)}\right)\to H_{n,q}\left(C^{\left(y\right)}\right)\right),\quad n\unicode{x2A7E} 0,\end{align*}

where rank of $H_{n,q}^{x,y}$ is the *q*th (*x*, *y*)-persistent Betti numbers [56].

### 4.2. Persistent Mayer Dirac

Given an embedding of *N*-chain complexes $\phi: (A_*, a) \longrightarrow (B_*, b)$, by abuse of notation, we denote $\phi_n(A_n)$ by *A_n_*. Let $1\unicode{x2A7D} q \unicode{x2A7D} N-1$, $0\unicode{x2A7D} i < N-q$ be integers. We compute an auxiliary chain complex $(C_{*,i,q}, c_{*,i,q})$, defined by setting first $C_{0,i,q} = B_i$, and for $n\unicode{x2A7E} 1$, it is given by

\begin{align*}C_{n,i,q} = \left(b^{q}_{t_n}\right)^{-1}\left(A_{t_{n-1}}\right)\end{align*}

as shown in the following diagram

**Figure:**
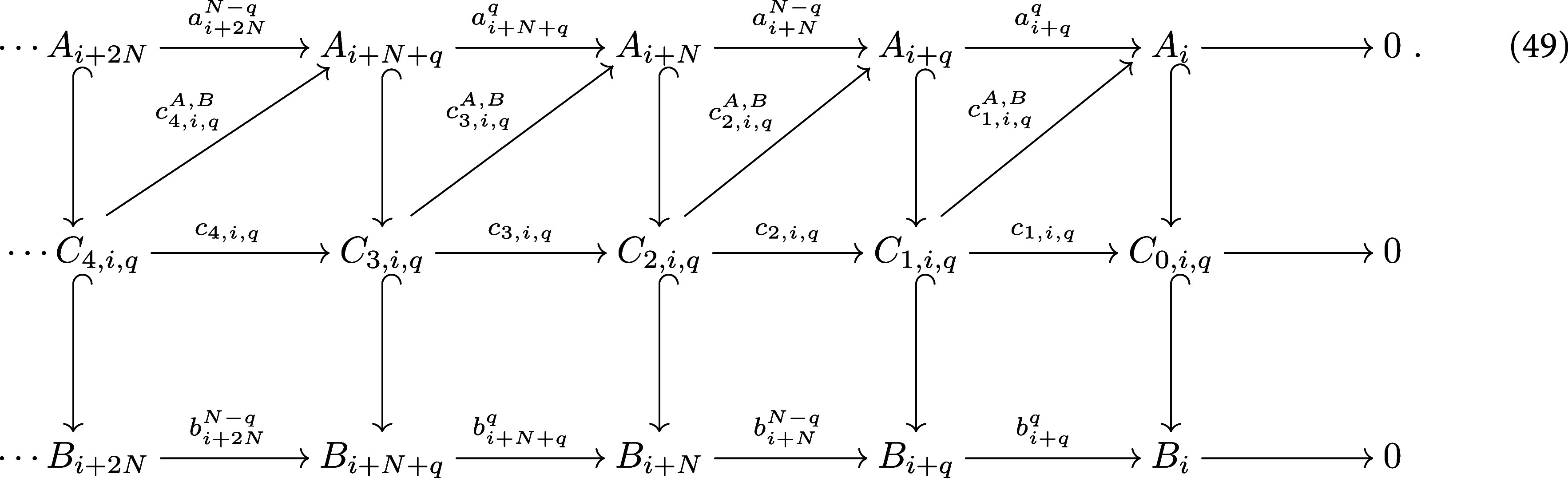


where

\begin{align*}t_n = \left\{ \begin{array}{ll} i+\frac{n-1}{2}N+q, &amp; n\ \hbox{is odd;} \\ i+\frac{n}{2}N, &amp; n\ \hbox{is even,} \end{array} \right.\end{align*}

and $c_{\ast,i,q}$ is the restriction of $b^q_{t_n}$. $\mathbf{c}_{\ast,i,q}$ is the matrix representation of $c_{\ast,i,q}$ with respect to a chosen orthonormal basis. For $k\unicode{x2A7E} 0$, the (*A*, *B*)-persistent Mayer Dirac operator $D^{A,B}_{k,i,q} $ is defined as the *k*th Dirac operator of the auxiliary complex shown above and is given by

\begin{align*} \mathbf{D}^{A,B}_{k,i,q} = \begin{pmatrix} \textbf{0}_{n_0\times n_0} &amp; \mathbf{c}_{1, i,q} &amp; \textbf{0}_{n_0\times n_2} &amp; \cdots &amp; \textbf{0}_{n_0\times n_{k}} &amp; \textbf{0}_{n_0\times n_{k+1}} \\ \mathbf{c}_{1, i,q}^* &amp; \textbf{0}_{n_1\times n_1} &amp; \mathbf{c}_{2, i,q} &amp; \cdots &amp; \textbf{0}_{n_1\times n_{k}} &amp; \textbf{0}_{n_1\times n_{k+1}} \\ \textbf{0}_{n_2\times n_0} &amp; \mathbf{c}_{2, i,q}^* &amp; \textbf{0}_{n_2\times n_2} &amp; \cdots &amp; \textbf{0}_{n_2\times n_{k}} &amp; \textbf{0}_{n_2\times n_{k+1}} \\ \vdots &amp; \vdots &amp; \vdots &amp; \ddots &amp; \vdots &amp; \vdots \\ \textbf{0}_{n_{k}\times n_0} &amp; \textbf{0}_{n_{k}\times n_1} &amp; \textbf{0}_{n_{k}\times n_2} &amp; \cdots &amp; \textbf{0}_{n_{k} \times n_{k}} &amp; \mathbf{c}_{k+1, i,q}\\ \textbf{0}_{n_{k+1}\times n_0} &amp; \textbf{0}_{n_{k+1}\times n_1} &amp; \textbf{0}_{n_{k+1}\times n_2} &amp; \cdots &amp; \mathbf{c}_{k+1, i,q}^* &amp; \textbf{0}_{n_{k+1}\times n_{k+1}} \end{pmatrix}.\end{align*}

On the other hand, the (*A*, *B*)-persistent Mayer Laplacian is defined as the persistent Laplacian of the following sequence at *A_n_* [56],

**Figure:**
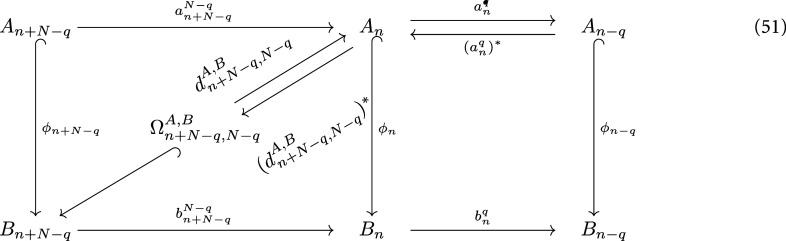


and is given by

\begin{align*} \Delta_{n,q}^{A,B}: = \left(a_{n}^q\right)^{\ast}\circ a_{n}^q+d_{n+N-q,N-q}^{A,B}\circ \left(d_{n+N-q,N-q}^{A,B}\right)^{\ast},\end{align*}

where

\begin{align*} \Omega_{n+N-q,N-q}^{A,B} = \left\{x\in B_{n+N-q}|b_{n+N-q}^{N-q}x\in A_{n}\right\},\quad 1\unicode{x2A7D} q\unicode{x2A7D} N-1,\end{align*}

and

\begin{align*} d_{n+N-q,N-q}^{A,B}v = b_{n+N-q}^{N-q}v.\end{align*}

In particular, if *n* < *q*, the persistent Mayer Laplacian is reduced to $\Delta_{n,q}^{A,B} = d_{n+N-q,N-q}^{A,B}\circ (d_{n+N-q,N-q}^{A,B})^{\ast}$.

We notice that every such sequence is involved in the construction of a unique auxiliary complex. As in the case of 2-chains, e.g. the traditional chain complexes, the harmonic component of the persistent Mayer Laplacian and persistent Mayer homology are also isomorphic [56]. That is,

\begin{align*}\ker \Delta_{n,q}^{A,B}\cong H_{n,q}^{A,B},\end{align*}

where $n\unicode{x2A7E} 0$ and $1\unicode{x2A7D} q\unicode{x2A7D} N-1$.

## 5. Applications

Recently, the advent of artificial intelligence has led to the proposal of numerous models aimed at encoding the chemical identity of a molecular entity through its chemical composition and atomic configuration. These encodings, called molecular descriptors or representations, play a crucial role in cheminformatics. Among them, molecular fingerprints are essential for virtual screening and mapping chemical space tasks. This section introduces models based on persistent Mayer Dirac operators, assessing their efficacy in providing topological and geometrical insights into molecules. The application involves computing complexes derived from molecules and evaluating various features associated with these complexes. Let $D: V \to V$ represent a Hermitian operator on a finite-dimensional vector space *V*, with $\lambda_1, \cdots, \lambda_k$ denoting its positive eigenvalues. The associated Dirac zeta function [35, 37] is defined as:

\begin{align*} \xi_D\left(s\right) = \sum_{i = 1}^k \lambda_i^{-s},\end{align*}

and its *m*th complexity [35, 38] is defined as:

\begin{align*} c_m\left(D\right) = \left(-1\right)^{km} \prod_{j = 1}^{k} \lambda_j^{2m}.\end{align*}

The advantage of Dirac zeta functions is that they can be used to distinguish manifolds that are cohomological and homotopically identical [37]. The number of the non-zero eigen pairs also called the (signless) Euler-Poincaré number [35, 38], is given by

\begin{align*} l\left(D\right) = \frac{1}{2}\dim\left(V\right) - \frac{1}{2}\ker\left(D\right),\end{align*}

and the spanning tree number [35] is defined as

\begin{align*} t\left(D\right) = \frac{1}{2}\log\left(|c_1\left(D\right)|\right) - \log\left(l\left(D\right)+1\right).\end{align*}

From the definition, *l*(*D*) tells us the number of pairs of eigenvalues of a Dirac operator, and it is an even number if and only the complexity of the operator is positive. Furthermore, in the case of graphs, the complexity is linked to graph connectivity, the existence of triangles, and the number of vertices. For instance, if a graph is connected, without triangles, and has an even number of vertices, then the complexity is negative [38]. The mean of the operator *D* is defined as

\begin{align*} \overline{\lambda}\left(D\right) = \frac{\xi_D\left(-1\right)}{k},\end{align*}

and its generalized mean is given by

\begin{align*}\hat{\lambda}\left(D\right) = \frac{\sum_{i = 1}^k |\overline{\lambda}\left(D\right)-\lambda_i|}{k}.\end{align*}

**Figure 3.**
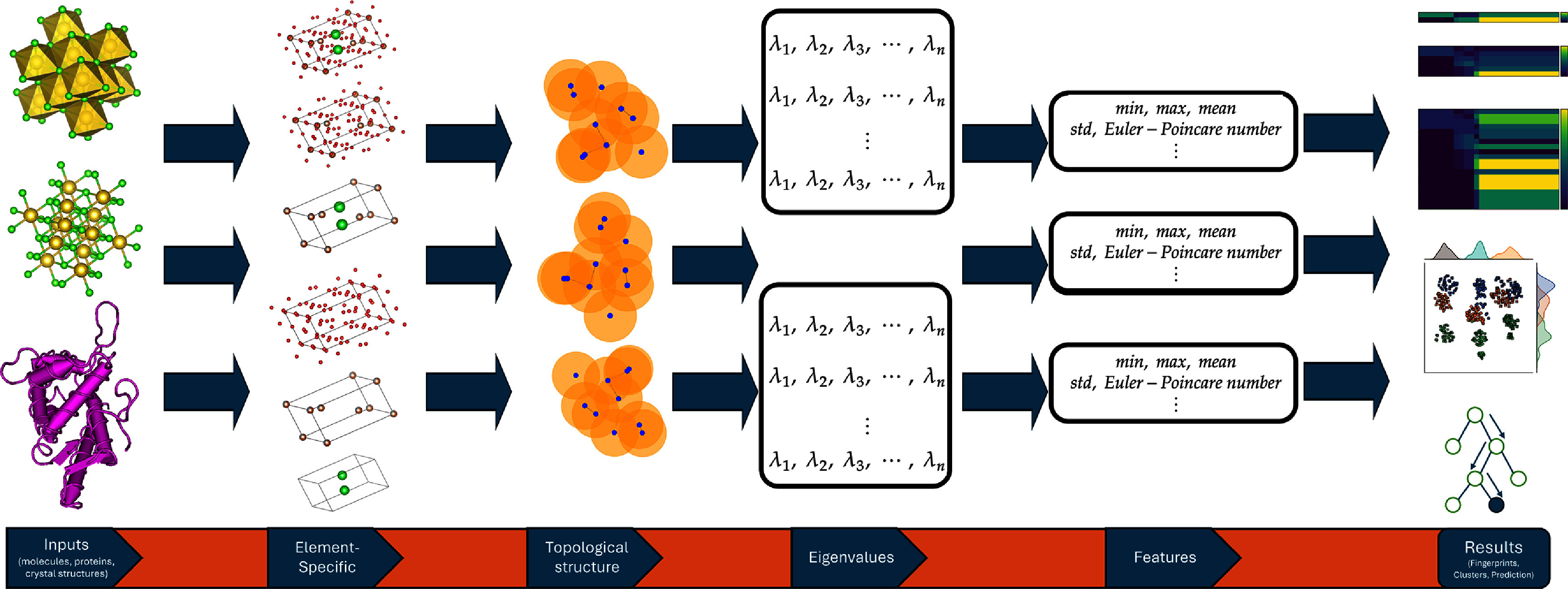
A proposed scheme of utilizing (Persistent) Mayer Dirac operators in biological and chemical applications.

This proves particularly beneficial when the eigenvalues deviate from their mean. Applying the generalized mean in the framework of mean absolute deviation eliminates the direct dependence of the cumulative absolute deviation of each eigenvalue from the mean eigenvalue on the size of the molecule. This methodology is elucidated in [25] and contributes to developing quantitative structural models for a diverse range of molecules [49, 52]. Our interest also extends to the attributes outlined in table 2 [35]. Figure 3 demonstrates a proposed scheme of utilizing (Persistent) Mayer Dirac operators in analyzing molecules.

**Table 2.** A table of persistent attributes extracted from persistent Mayer Dirac operators.

Persistent Attributes and Features Extracted			
Attribute/Feature	Description	Attribute/Feature	Description
$\eta(D)$	The nullity of the Dirac operator.	$c_m(D)$	Complexity.
$ \overline{\lambda}(D) $	The average of the positive eigenvalues of the Dirac operator.	*l*(*D*)	The (signless) Euler-Poincare number.
$\hat{\lambda}(D)$	The generalized mean.	*t*(*D*)	Spanning tree.
$\xi(-1)$	Laplacian graph energy.	$\textrm{min}\{\lambda_1,\cdots, \lambda_k\}$	
$\xi(-2)$	Spectral second moment.	$\textrm{max}\{\lambda_1,\cdots, \lambda_k\}$	
$\xi(2)$		The standard deviation	
$(k+1)\xi(1)$	Quasi-Wiener Index.		

### 5.1. Persistent Mayer Dirac characterization of isomers

**Figure 4.**
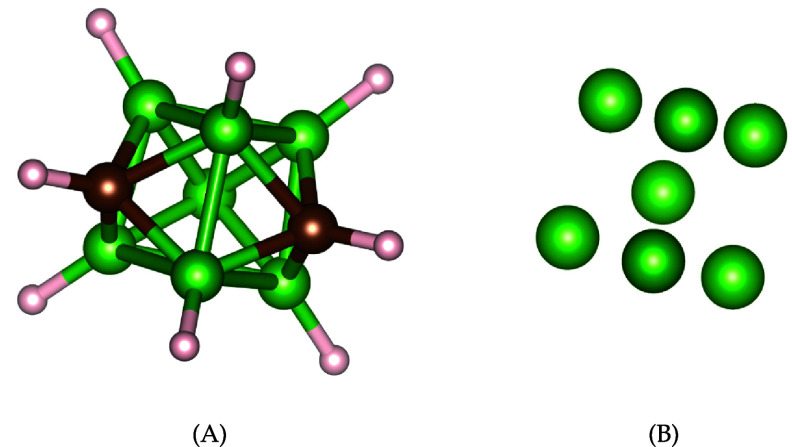
(A) The first isomer of of $\textrm{B}_7\textrm{C}_2\textrm{H}_9$. (B) Only boron atoms of the first isomer.

**Figure 5.**
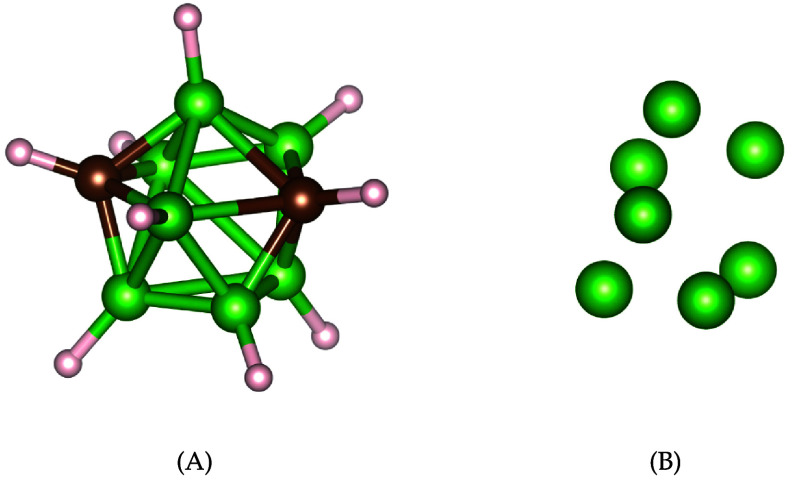
(A) The second isomer of of $\textrm{B}_7\textrm{C}_2\textrm{H}_9$. (B) Only boron atoms of the second isomer.

**Figure 6.**
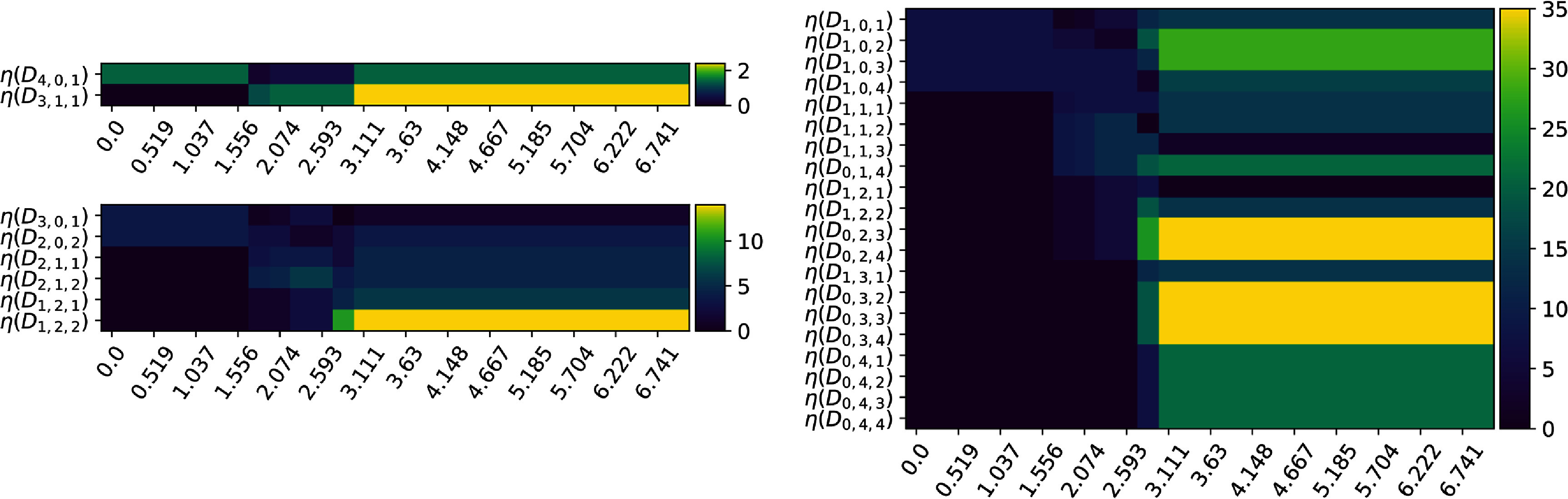
Nullities of a family of persistent Mayer Dirac operators of the first isomer as the filtration parameter increases. The plots from smallest to largest correspond to *N* = 2, *N* = 3 and *N* = 5 respectively.

**Figure 7.**
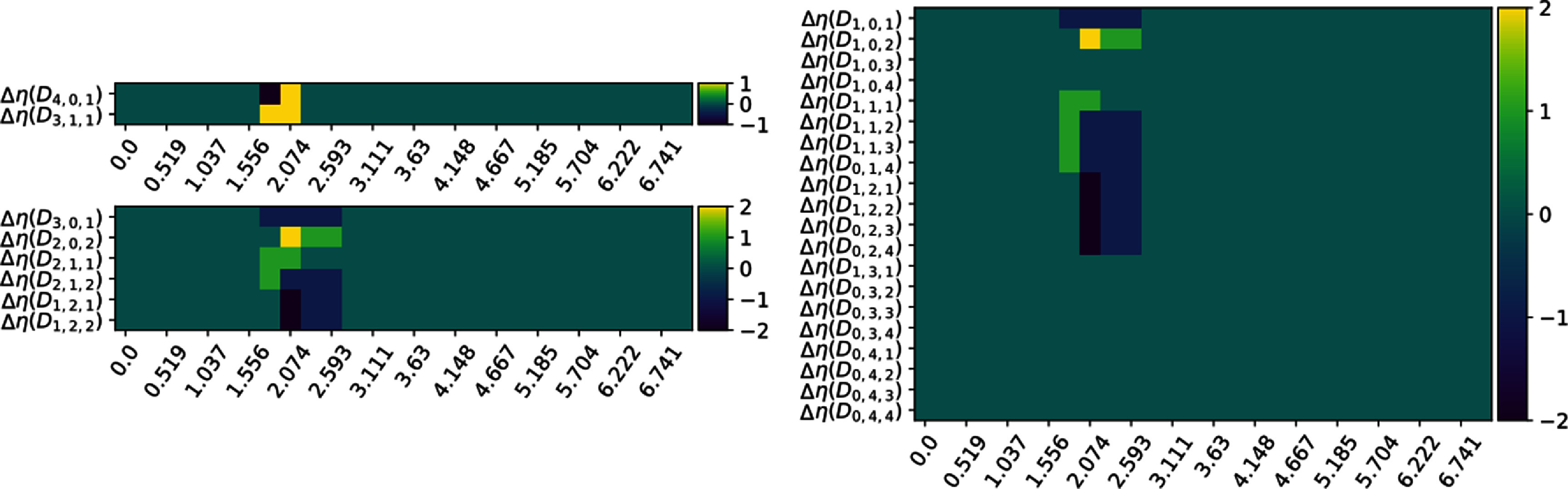
The difference of the nullities of persistent Mayer Dirac operators of the first and second isomers as the filtration parameter increases. The plots from smallest to largest correspond to *N* = 2, *N* = 3 and *N* = 5 respectively. Apparently, the width of the difference is larger in the cases of *N* = 3 and *N* = 5 since Mayer homology tells simplicies of different dimensions apart.

One intriguing aspect of organic chemistry lies in its three-dimensional nature. The spatial configuration of a molecule plays a crucial role in determining its properties. Variations in the arrangement of atoms contribute to the existence of isomers—different assemblies of the same combination of atoms. Isomers share identical molecular formulas but exhibit distinct atom arrangements. Differentiating between isomers involves various techniques, including examining physical properties such as melting point, boiling point, density, refractive index, and others [55]. Various methods, such as graph theory and topological approaches, aid in distinguishing isomers [20, 59, 63]. In this context, we propose a topological approach utilizing Mayer homology and Dirac operators. The persistent Mayer Dirac operators effectively assign unique characteristics to different isomers. For specific isomers, where atoms exhibit different spatial positions, Vietoris-Rips filtration on molecules can be employed for differentiation. However, in cases where atoms maintain the same relative positions across isomers, an element-specific approach can be valuable [14]. This technique, treating each type of atom independently, has proven powerful in various applications. We apply persistent Mayer Dirac operators with a specific-element approach [15], utilizing a filtration on each type of atom within the molecules. To illustrate, we examine two isomers of $\textrm{B}_7\textrm{C}_2\textrm{H}_9$ carborane, composed of borons, carbons, and hydrogens depicted in figure 4 and figure 5. Applying Vietoris-Rips filtration on boron atoms for each isomer, we calculate their Dirac operators’ eigenvalues and analyze their features. The use of Vietoris-Rips simplicial complexes with a filtration based on interatomic distances, ranging from 0 Å to 7 Å, reveals noticeable differences in fingerprints obtained from boron atoms as illustrated in figure 6. While boron atom fingerprints show minimal disparities when *N* = 2, the contrast in features between the two isomers becomes evident when *N* > 2, for instance, *N* = 3 and *N* = 5 as illustrated in figure 7.

### 5.2. Organic-inorganic halide perovskites (OIHP) characterization

**Figure 8.**
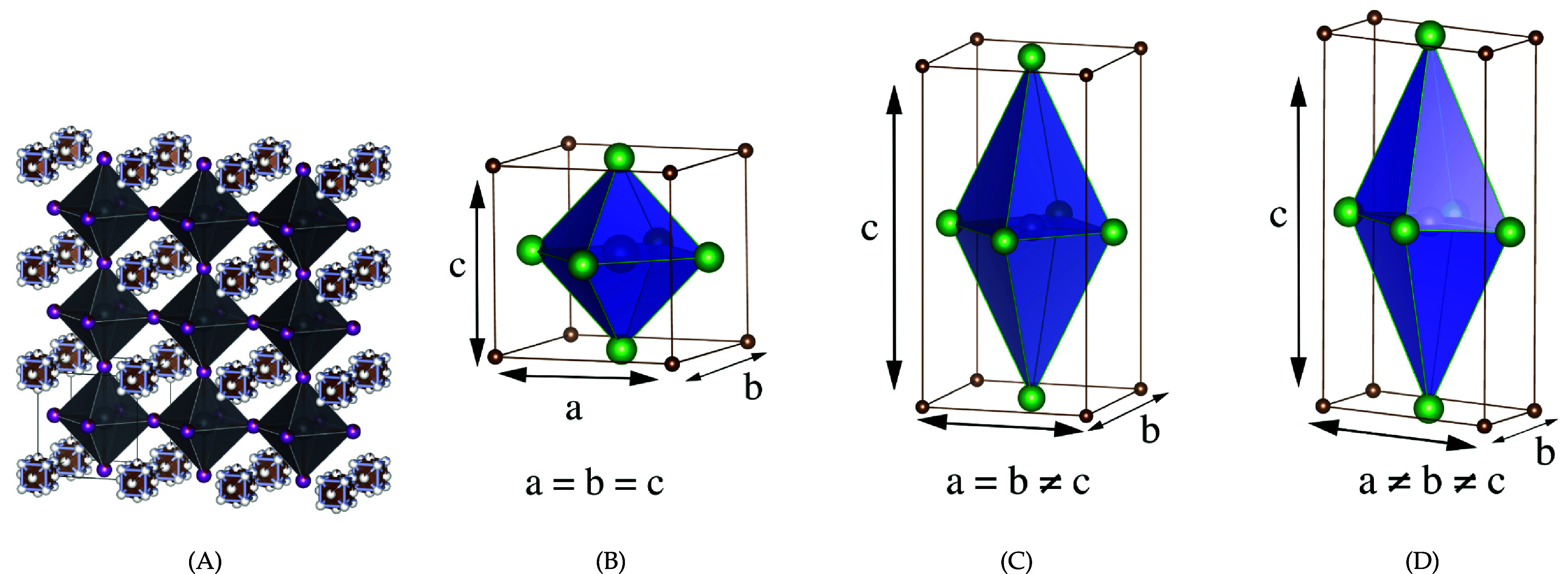
(A) A general representation of the crystal structures of OIHP materials considered, where the image depicts only a part of a single layer. (B) The unit of the cubic configuration. (C) The unit of the tetragonal configuration. (D) The unit of the orthorhombic configuration.

**Figure 9.**
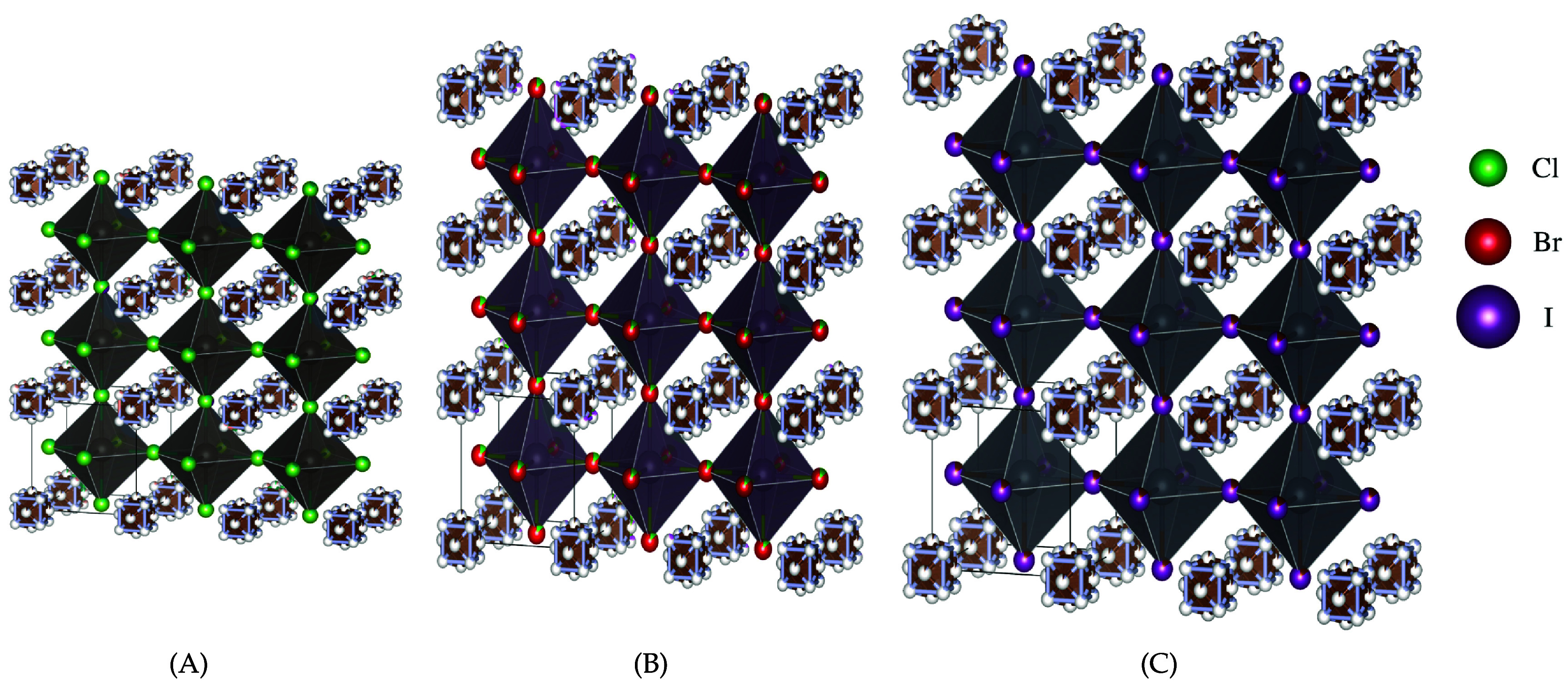
(A) The unit size of $\textrm{C(NH}_2)_3\textrm{SnCl}_3$, in general. (B) The unit size of $\textrm{C(NH}_2)_3\textrm{SnBr}_3$, in general. (D) The unit size of $\textrm{C(NH}_2)_3\textrm{SnI}_3$, in general.

**Figure 10.**
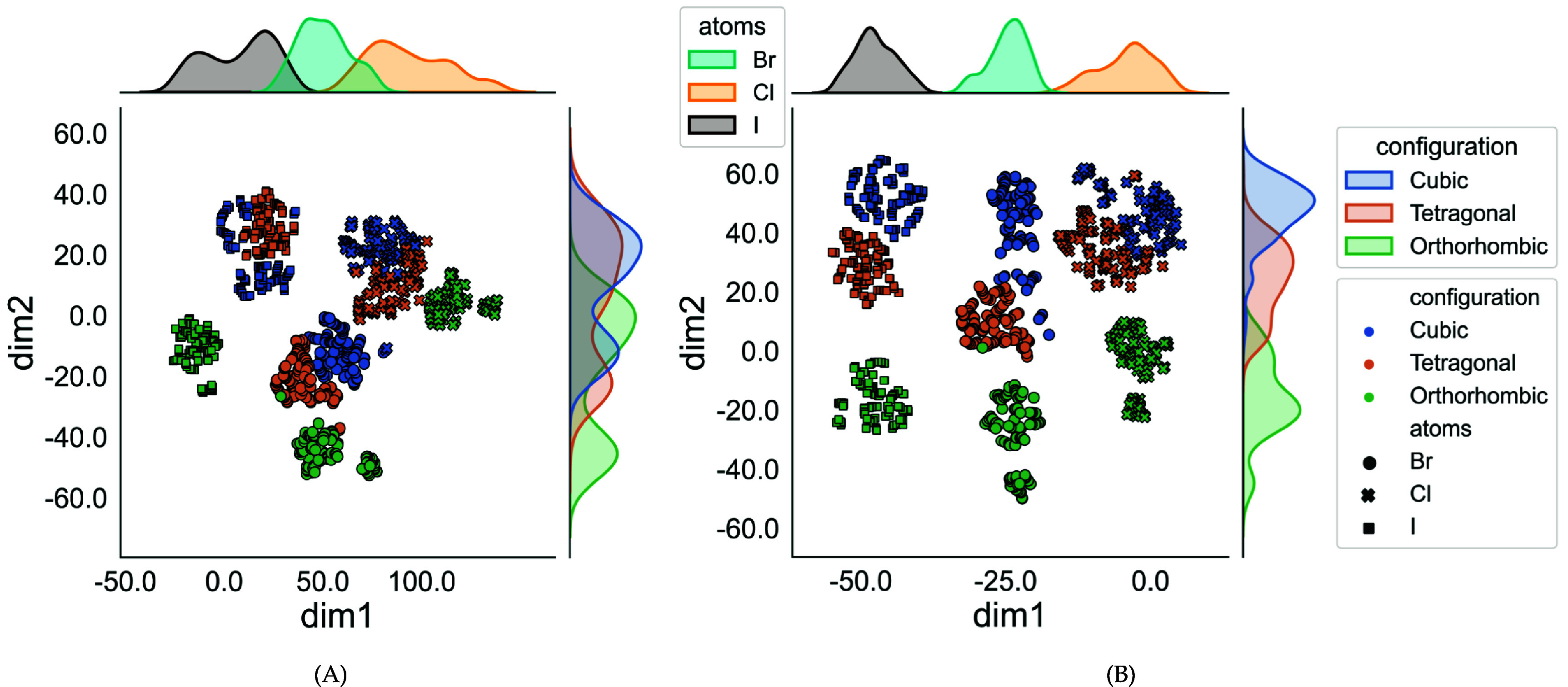
The t-SNE visualization of organic-inorganic halide perovskites (OIHP) represented by weighted and unweighted persistent Mayer Dirac operators. (A) The clusters resulted from unweighted persistent Mayer Dirac operators, with *N* = 5. (B) The clusters resulted from weighted persistent Mayer Dirac operators, with *N* = 5. The upper kernel density plot is labeled by the atom types, while the left one is labeled by the configuration type.

Metal halide perovskites, whether organic or inorganic, present economical, highly processable, and performance -efficient materials with significant potential in photovoltaic applications [3, 7, 42, 51]. These materials exhibit crystal structures depicted in images, undergoing temperature-dependent transformations from cubic to tetragonal and orthorhombic. Organic/inorganic metal halide perovskites (OIHPs) generally conform to the ABX3 crystal structure, where A represents a monovalent cation (either organic or inorganic, such as Cs or $\textrm{CH}_3\textrm{NH}_3-$), B is a divalent cation (e.g. Pb, Sn). X is a halide anion (e.g. Cl, I, Br). TDA methods and machine learning algorithms have displayed promising outcomes in characterizing these materials [4, 35]. This study demonstrates that persistent Mayer Dirac operators offer intricate structural insights into OIHPs, forming a foundation for understanding their physical properties and functionalities. Notably, this analysis solely relies on geometric information, such as atomic positions, indicating its sufficiency for characterizing OIHP materials. The investigation employs three Methylammonium lead halides (MAPbX3) variants—where X represents Cl, Br, or I—in three distinct phases: orthorhombic, tetragonal, and cubic [35]. Each type encompasses 300 samples, with a hundred for each phase, resulting in 900 samples. The configuration of each type is illustrated in figure 8 according to [3]. The difference of the lengths based on the atom type *X* in the case of the orthorhombic configuration is shown in figure 9 based on [51]. Treating atoms as points in space, the study generates a sequence of Vietoris-Rips simplices and applies Mayer homology. The filtration radius ranges from 0 Å to 7 Å, resulting in a sequence of 28 *N*-chain complexes with *N* values of 3 and 5 for comparative analysis. Both weighted and unweighted approaches are tested, with weights for the square of the average distance between points in a simplex and equal weights for vertices. After calculating features, the t-SNE algorithm is employed to reduce dimensionality for visualization. The results reveal a division of samples into three major components, each corresponding to a specific type of OIHP as illustrated in figure 10. Additionally, each component occupies a bandwidth on the first dimension based on the atomic number, with the left cluster corresponding to iodine (highest atomic number), followed by bromine and then chlorine. Further subdivision within each significant component based on sample geometry (cubic, tetragonal, or orthorhombic) is observed. The study concludes that persistent Mayer Dirac operators successfully capture the geometric information of OIHPs, characterizing them effectively and demonstrating continuity based on the provided geometric information.

## 6. Conclusion

This study explores Mayer homology as a tool for discerning simplices of varying dimensions, which assigns unique Mayer Betti numbers to each simplex of different dimensions. It holds promise as a valuable instrument for capturing the intrinsic geometric properties of data. The study thoroughly examines Mayer *N*-chain complexes, Mayer homologies, Laplacians, and Dirac operators. It lays the groundwork for a profound understanding of these mathematical structures. It investigates innovative concepts, including Mayer Laplacians and Mayer Dirac operators. The study revisits the definitions of Mayer *q*th *n*-cycles, boundaries, and homology groups and presents classical examples. A table detailing non-zero Mayer homological groups associated with the first few simplices is also presented. The study extends to practical applications, introducing weighted versions of Laplacian and Dirac operators and demonstrating their effectiveness in capturing physical attributes in diverse scenarios. The formulation of persistent Mayer Laplacian and Mayer Dirac operators is also presented, specifically emphasizing applications in biological and chemical domains, particularly in analyzing molecular structures. The study proposes applications of Mayer Dirac operators, suggesting their efficacy as fingerprints for molecular distinctions. Comparative analysis with classical counterparts reveals that Mayer Dirac operators offer richer insights, showcasing a broader bandwidth that enhances molecule differentiation. In essence, this work contributes to the theoretical comprehension of Mayer complexes and opens avenues for practical applications. It underscores the potential of Mayer Dirac operators to advance our understanding of complex structures across diverse scientific fields.

## Data Availability

The data that support the findings of this study are openly available at the following URL/DOI: https://github.com/FaisalSuwayyid/persistent-Mayer-Dirac.
